# The rotation of primary starter culture mixtures results in batch-to-batch variations during Gouda cheese production

**DOI:** 10.3389/fmicb.2023.1128394

**Published:** 2023-02-16

**Authors:** Hannes Decadt, Stefan Weckx, Luc De Vuyst

**Affiliations:** Research Group of Industrial Microbiology and Food Biotechnology (IMDO), Faculty of Sciences and Bioengineering Sciences, Vrije Universiteit Brussel, Brussels, Belgium

**Keywords:** starter culture rotation, metabolomics, cheese ripening, Gouda cheese core, Gouda cheese rind, high-throughput full-length 16S rRNA gene sequencing

## Abstract

Industrial production of Gouda cheeses mostly relies on a rotated use of different mixed-strain lactic acid bacteria starter cultures to avoid phage infections. However, it is unknown how the application of these different starter culture mixtures affect the organoleptic properties of the final cheeses. Therefore, the present study assessed the impact of three different starter culture mixtures on the batch-to-batch variations among Gouda cheeses from 23 different batch productions in the same dairy company. Both the cores and rinds of all these cheeses were investigated after 36, 45, 75, and 100 weeks of ripening by metagenetics based on high-throughput full-length 16S rRNA gene sequencing accompanied with an amplicon sequence variant (ASV) approach as well as metabolite target analysis of non-volatile and volatile organic compounds. Up to 75 weeks of ripening, the acidifying *Lactococcus cremoris* and *Lactococcus lactis* were the most abundant bacterial species in the cheese cores. The relative abundance of *Leuconostoc pseudomesenteroides* was significantly different for each starter culture mixture. This impacted the concentrations of some key metabolites, such as acetoin produced from citrate, and the relative abundance of non-starter lactic acid bacteria (NSLAB). Cheeses with the least *Leuc. pseudomesenteroides* contained more NSLAB, such as *Lacticaseibacillus paracasei* that was taken over by *Tetragenococcus halophilus* and *Loigolactobacillus rennini* upon ripening time. Taken together, the results indicated a minor role of leuconostocs in aroma formation but a major impact on the growth of NSLAB. The relative abundance of *T. halophilus* (high) and *Loil. rennini* (low) increased with ripening time from rind to core. Two main ASV clusters of *T. halophilus* could be distinguished, which were differently correlated with some metabolites, both beneficial (regarding aroma formation) and undesirable ones (biogenic amines). A well-chosen *T. halophilus* strain could be a candidate adjunct culture for Gouda cheese production.

## 1. Introduction

Gouda cheese production is a complex interplay of a mixed-strain starter culture, non-starter microorganisms, and time. A typical Gouda cheese starter culture, used in lyophilized or frozen form, contains (undefined) strains of the lactic acid bacteria (LAB) *Lactococcus lactis* subsp. *lactis* (*Lc. lactis*), *Lactococcus cremoris* subsp. *cremoris* (*Lc. cremoris*), *Lactococcus lactis* subsp. *lactis* biovar diacetylactis (*Lc. lactis* biovar diacetylactis), and *Leuconostoc mesenteroides* and/or *Leuconostoc lactis* (Düsterhöft et al., 2017). The main role of this mesophilic starter culture is to lower the pH of the fermenting milk by converting lactose into lactate, which is almost completed after 10 h. Besides acidification by the homofermentative *Lactococcus*, citrate utilization by *Lc. lactis* biovar diacetylactis and/or leuconostocs occurs, which results into buttery flavor compounds, such as diacetyl, acetoin, and/or 2,3-butanediol (Starrenburg and Hugenholtz, 1991; Smit et al., 2005; Steele et al., 2013; Düsterhöft et al., 2017). In addition, all strains contribute to flavor development through proteolysis, peptidolysis, and amino acid conversions. The heterofermentative leuconostocs produce few eyes because of carbon dioxide formation through heterolactic fermentation and citrate conversion. These subprocesses of fermentation and ripening determine the complex and dynamic flavor profile of Gouda cheese, which is characterized by a sweet and mild taste.

The application of the same LAB starter cultures in a Gouda cheese production environment increases the risk of a bacteriophage infection, which may lead to failed fermentation processes and thus economical losses (Mahony and van Sinderen, 2014). Starter cultures consisting of a complex mixture of strains are in general less phage-sensitive, but a starter culture with only few strains guarantees a more consistent end-product quality (Erkus et al., 2013; Spus et al., 2015). Therefore, most cheese factories apply a starter culture rotation with three to five starter cultures with different phage sensitivity profiles (Høier et al., 2010; Parente et al., 2017; Frantzen et al., 2018). However, little is known about the impact of the use of rotating starter cultures on the final organoleptic properties of Gouda cheese throughout various batch productions.

Although mixed-strain LAB starter cultures are used to steer the Gouda fermentation and ripening process, mesophilic non-starter lactic acid bacteria (NSLAB) originate from the milk or the cheese production equipment or environment (e.g., brine bath and ripening room) and are able to grow to counts as high as 10^8^ CFU/g during the ripening phase (Fitzsimons et al., 2001; Van Hoorde et al., 2008; Fox et al., 2017; Gobbetti et al., 2018). In general, *Lacticaseibacillus paracasei* and *Lactiplantibacillus plantarum* are the main NSLAB across various cheese types ripened for 4 months or longer, including Gouda cheese (Gobbetti et al., 2015). Positive contributions of NSLAB to cheese flavor are well known, as they hydrolyze peptides and convert the resulting free amino acids into flavor compounds, which however depends on the production batch (Montel et al., 2014; Gobbetti et al., 2015). Moreover, in some cases, they cause off-flavors or undesired gas production (Martley and Crow, 1993; Oberg et al., 2016). To take benefit from the NSLAB regarding flavor formation, but to avoid batch variability, selected NSLAB are added as adjunct cultures in some cheese productions (Antonsson et al., 2003; Van Hoorde et al., 2010b; O’Brien et al., 2017; Randazzo et al., 2021). Additionally, *Enterococcus durans*, *Enterococcus faecalis*, *Enterococcus faecium*, *Lacticaseibacillus rhamnosus*, *Latilactobacillus curvatus*, *Lentilactobacillus parabuchneri*, and *Pediococcus pentosaceus* may be part of the Gouda cheese NSLAB communities (Van Hoorde et al., 2008, 2010a). Also, culture-dependent methods detect a higher relative abundance of NSLAB in aged Gouda cheese compared to culture-independent approaches, because the starter culture strains occur in a viable but not culturable (VBNC) state during ripening that can only be detected through DNA sequencing (Ganesan et al., 2007; Ruggirello et al., 2014). Finally, given the reclassification of the *Lactobacillus* genus (Zheng et al., 2020), an update is needed to assess the full diversity of lactobacilli in Gouda cheese.

Whereas culture-dependent methods are more laborious and only isolate microorganisms that can grow on the agar media and under the incubation conditions applied, resulting into an inherent selection bias, culture-independent methods are more suited to investigate the taxonomical diversity of a microbial ecosystem (Ercolini, 2013; De Filippis et al., 2017; Levante et al., 2021). Most metagenetic high-throughput amplicon-based sequencing studies on cheese target only a part of the 16S rRNA gene, as has been performed, for instance, for Irish artisan cheeses (Quigley et al., 2012), soft Herve cheese (Delcenserie et al., 2014), Portuguese Pico cheese (Riquelme et al., 2015), traditional Brazilian cheeses (Kamimura et al., 2019), Italian mozzarella (Marino et al., 2019), Gouda cheeses (Park et al., 2019), and Grana Padano cheeses (Zago et al., 2021). Alternatively, the microbiota of some cheeses has been investigated by high-throughput sequencing of the whole 16S rRNA gene, using PacBio technology, which allows a more accurate species-level identification (Jin et al., 2018; Yang et al., 2020). The latter study has also attempted to link the most prevailing species with sensory profiles generated with an electric nose (Yang et al., 2020). However, since the metagenetic sequence reads were clustered into operational taxonomic units (OTUs) instead of amplicon sequence variants (ASVs), taxonomic assignment could not be performed below species level (Callahan et al., 2017). Indeed, ASVs allow a single-nucleotide resolution that can resolve taxonomical identification at species and sometimes even strain level (Callahan et al., 2019; Johnson et al., 2019). In that perspective, a meta-analysis of cheese microbiomes has indicated a significantly different volatile profile for strains of the same species, encompassing *Brevibacterium linens*, *Lc. lactis*, and *Streptococcus thermophilus* involved in artisan Irish cheese production (Walsh et al., 2020), which emphasizes the relevance of obtaining a reliable taxonomical resolution below species level.

Besides an accurate taxonomic assignment of the microbial dynamics during Gouda cheese ripening, a thorough metabolomic analysis as a function of the ripening time is needed, in particular to be able to make the link between the microbial taxa and flavor compounds produced unequivocally. For example, research on smear-ripened cheddar cheeses has correlated both *Debaryomyces hansenii* and *Glutamicibacter arilaitensis* with alcohols and carboxylic acids, and the latter species with ketones as well, whereas *B. linens* and *Geotrichum candidum* have been correlated with sulfur compounds (Bertuzzi et al., 2018). An in-depth study of Gouda cheese has investigated the sensory and chemical properties of 36 productions from five countries and indicated the age of the cheeses as most determining factor to explain differences in concentrations of flavor compounds formed during ripening (Jo et al., 2018). Lactic acid has been identified as the main organic acid in Gouda cheeses. Acetic acid, diacetyl, 2-and 3-methylbutanal, and ethyl butyrate are among the most important aroma-active compounds. This is in line with other studies that have indicated acetoin (buttery notes), lactones (fruity/sweet/buttery notes), methylketones (floral/nutty notes), and short-chain fatty acids (cheesy/rancid notes) as additional major constituents of Gouda cheeses (Dirinck and De Winne, 1999; Van Leuven et al., 2008; Van Hoorde et al., 2010b; Jung et al., 2013; Ruyssen et al., 2013). Sulfur compounds, such as dimethyl sulfides and methional, are present at relatively low concentrations, but do have an impact on the Gouda cheese flavor (Van Leuven et al., 2008; Jo et al., 2018). In addition to their contribution to the basic cheese taste (Toelstede and Hofmann, 2008), amino acids are precursors for volatile organic compounds that contribute to the cheese aroma, such as higher alcohols, higher aldehydes, and esters (Smit et al., 2005). However, they can also be the precursors of biogenic amines, which have a toxicological potential, especially histamine and tyramine (Komprda et al., 2007; Linares et al., 2012). All these compounds can be produced by a wide range of LAB species, which is considered to be a strain-dependent trait (Barbieri et al., 2019; Walsh et al., 2020). However, few studies have focused on potential differences in microbial and metabolite compositions of the core and rind of the cheeses investigated (Calasso et al., 2016; Dugat-Bony et al., 2016; Salazar et al., 2018).

Whereas several studies on Gouda cheese have focused on its microbial composition with limited focus on the metabolites produced, other studies have investigated a restricted part of the metabolome with limited attention for the microbial composition. Therefore, the present study aimed at a systematic assessment of the overall microbiome (below species level) and metabolome (including endogenous and microbial substrates and metabolites, both volatile and non-volatile ones) as well as their link with the application of rotated starter cultures and/or cheese cores and rinds of Gouda cheeses from 23 different production batches, throughout their whole ripening time and made in the same dairy factory.

## 2. Materials and methods

### 2.1. Cheese production, selection, and sampling

**Figure 1:**
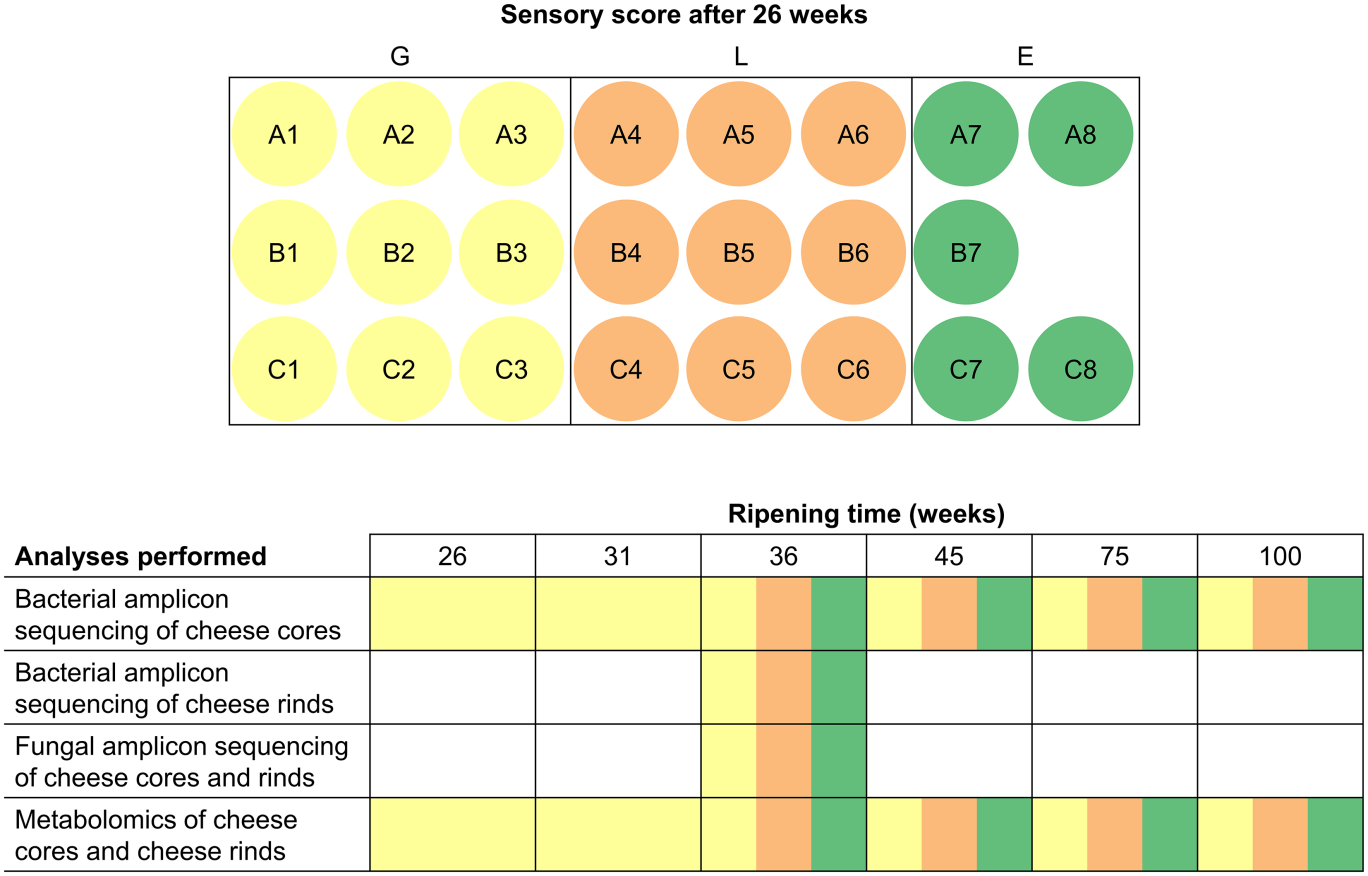
Overview of all cheese batch productions investigated during the present study and the multiphasic analyses performed. Each letter (A, B, and C) indicates the starter culture mixture applied that was used in a rotation system. The color indicates the sensory score given after 26 weeks of ripening: yellow, good (G); orange, less than average (L); green, exceptionally good (E). All cheese batch productions were investigated after 36, 45, 75, and 100 weeks of ripening. Wheels from batches with sensory score G were additionally investigated after 26 and 31 weeks of ripening.

Gouda cheeses (23 production batches) were produced in a European dairy factory between mid-July and mid-August 2018 from standardized and pasteurized milk, according to a standard Gouda manufacturing process (Düsterhöft et al., 2017), using three different, commercial, frozen, mesophilic, undefined, mixed-strain LAB starter cultures (A, B, and C), composed of *Lc. lactis*, *Lc. cremoris*, *Lc. lactis* biovar diacetylactis, and *Leuconostoc* spp. As these starter culture mixtures were used in a rotation system, the corresponding Gouda cheeses are further referred to as Gouda A (8 production batches, numbered A1–A8), B (7, B1–B7), and C (8, C1–C8) cheeses (Figure 1). All starter culture mixtures were from the same starter culture manufacturer. Cheeses of each batch production were organoleptically assessed by the company’s tasting panel after 26 weeks of ripening and scored as less than average (L), good (G), or exceptionally good (E). For further analysis, for each starter culture used, three cheese wheels with sensory score L and three wheels with sensory score G of the different production batches were selected, resulting in 18 cheese wheels. Additionally, five cheese wheels with sensory score E were selected, irrespective of the starter culture used. This selection resulted in a total of 23 Gouda cheese production batches for metagenetic and metabolomic analysis, which was carried out as a function of the ripening time. The analyses were represented by cheeses of a ripening time ready for sale (36 and 45 weeks) and long-ripened cheeses (75 and 100 weeks). Batches with sensory score G were also analyzed after 26 (time point of sensory assessment) and 31 weeks of ripening.

All selected cheese wheels were vacuum-packed and stored at 4°C in the factory before their transport to and further sampling in the research group’s laboratory. Therefore, a cheese wheel was cut into two halves and a slice of 3 cm from the border of one halve was taken. Three pieces of approximately 40 g of the core were cut out aseptically from this slice, which were further cut into smaller pieces, and then pooled. For the rind samples, pieces of approximately 60 g of the top and the bottom of the remaining halve were pooled. Ten grams of pooled sample, either core or rind, were transferred to a stomacher bag (Seward, Worthing, West Sussex, United Kingdom) and mixed at medium speed with 90 mL of a sterile sodium citrate solution (2%, m/v; Sigma-Aldrich, St. Louis, MO, United States) in a stomacher (Laboratory Blender Stomacher 400, Seward) for 5 min. This suspension was centrifuged at 4,500 × *g* for 20 min and the cell pellets obtained were divided into four aliquots. The pellets were resuspended in 1 mL of sorbitol buffer [1.2 M sorbitol (VWR International, Darmstadt, Germany) and 50 mM Tris-base (Calbiochem, San Diego, CA, United States); pH 7.5], centrifuged at 6,000 × *g* for 10 min, and the resulting cell pellets were stored at −25°C before further DNA extraction.

### 2.2. Metagenetic analysis

#### 2.2.1. DNA extraction

Whole-community DNA extraction of the cheese samples was performed as described previously (Vermote et al., 2018). Briefly, the cell pellets obtained as mentioned above were submitted to an enzymatic digestion [one incubation step with lyticase (Sigma-Aldrich) and Zymolyase (G-Biosciences, St. Louis, MO, United States) and another one with lysozyme (Merck, Darmstadt, Germany) and mutanolysin (Sigma-Aldrich)], a chemical/enzymatic treatment [with sodium dodecyl sulfate (Sigma-Aldrich) and proteinase K (Merck)], and a mechanical disruption by vortexing in the presence of acid-washed glass beads (Sigma-Aldrich), followed by protein removal using a mixture of chloroform, phenol and isoamyl alcohol (Sigma-Aldrich), a treatment with RNase (Roche, Basel, Switzerland), and DNA purification with a DNeasy Blood & Tissue Kit (Qiagen, Venlo, Netherlands). The DNA purity was assessed using a NanoDrop 2000 spectrophotometer and the DNA concentrations were measured with a Qubit 2.0 fluorometer using a Qubit dsDNA HS Assay Kit (all from Thermo Fisher Scientific, Carlsbad, CA, United States).

#### 2.2.2. Metagenetics

To assess the bacterial members represented by the whole-community DNA isolated from the cheese samples, amplicon-based high-throughput sequencing was applied for the cheese core samples taken at all time points as well as for the cheese rind samples taken after 36 weeks of ripening. Therefore, the bacterial full-length 16S rRNA gene was amplified with the universal primer set 27F (AGRGTTYGATYMTGGCTCAG) and 1492R (RGYTACCTTGTTACGACTT) (Integrated DNA Technologies, Leuven, Belgium). Both primers were tagged with 5′ sample-specific barcodes to allow for multiplexed sequencing, according to the manufacturer’s instructions (PacBio, Menlo Park, CA, United States). PCR assays were performed using the KAPA HiFi DNA Polymerase (Hot Start and Ready Mix formulation; Roche), with 3 min of denaturation at 95°C, followed by 24 cycles of denaturation at 95°C for 30 s, annealing at 57°C for 30 s, and extension at 72°C for 60 s. Post-amplification quality control was performed by capillary electrophoresis, using a Bioanalyzer 2100 and a High Sensitivity DNA Kit (Agilent Technologies, Santa Clara, CA, United States). The DNA concentrations were measured with a Qubit 2.0 fluorometer. The amplicon sets were equimolarly pooled, a circular sequencing adaptor was ligated, and the resulting amplicon library was sequenced using a PacBio Sequel system in circular consensus mode (VIB Nucleomics Core Facility, Leuven, Belgium). Circular consensus sequences (CCS) were generated, using SMRT Link 4.0 (PacBio), with the minimum predicted accuracy set to 0.998. These long-read sequences were clustered into ASVs with the DADA2 R software package (version 1.14.1), updated for long amplicon reads (Callahan et al., 2019). The filtering parameter settings were minQ = 3, minLen = 1100, maxLen = 1600, maxN = 0, and maxEE = 2. In the dada function, the pool parameter was set to “pseudo” to perform the analysis twice. First, all samples were processed by the DADA2 algorithm independently and all ASVs obtained were then used as input for the second analysis, during which the algorithm considered all ASVs previously found as true ASVs. Chimeras were removed, using the removeBimeraDenovo function, with the parameter settings minFoldParentOverAbundance = 3.5 and method = “consensus.” Taxonomy was assigned with the SILVA database (version 138; Quast et al., 2013), using the assignTaxonomy function with the parameter minBoot = 50. Taken the recent elevation of *Lc. lactis* subsp. *cremoris* to the species level as *Lactococcus cremoris* (Li et al., 2021) into account, all *Lactococcus* ASVs were compared with the type strains, *Lc. lactis* ATCC 19435^T^ and *Lc. cremoris* ATCC 19257^T^, and assigned to one of these species when the sequence identity was above 99.80%, or to *Lactococcus lactis/cremoris* otherwise.

To assess the fungal members represented by the whole-community DNA in the core and rind samples of the cheeses after 36 weeks of ripening, amplification of the internal transcribed spacer (ITS1) region of the fungal rRNA transcribed unit was performed with primer pair BITS1-B58S3 (Bokulich and Mills, 2013b). Further steps were performed using short-read sequencing (Illumina, San Diego, CA, United States) and sequence data analysis using DADA2, as described previously (De Bruyn et al., 2017). This allowed the identification at genus level. Only the forward reads were used, since the variable length of the ITS1 region did not allow a correct merging of the forward and reverse reads for all genera. The parameter settings maxN = 1, truncQ = 2, maxEE = 5, and minLen = 50 were applied. Taxonomy was assigned with the UNITE database (version 04.02.2020; Kõljalg et al., 2013).

All taxon data reported below are expressed as relative abundances of the total number of sequence reads.

#### 2.2.3. Starter cultures

The original starter culture mixtures were used for whole-community DNA extraction and plating. Therefore, 200 g of frozen starter culture was resuspended in a stomacher bag with 1800 mL of a sterile saline solution (8.5 g/L of NaCl; Merck). This suspension was centrifuged to yield cell pellets, which were subjected to DNA extraction, followed by amplicon-based high-throughput sequencing of the full-length 16S rRNA gene (PacBio) and DADA2 processing, as described above.

Given the fact that very few sequence reads of leuconostocs were found, a plating of the starter culture mixture was carried out to obtain *Leuconostoc* isolates. Therefore, serial dilutions of the suspensions mentioned above were made with sterile saline, and 100 μL of each dilution was plated on MRSv agar [de Man-Rogosa-Sharpe (MRS) agar (Oxoid, Basingstoke, Hampshire, United Kingdom) supplemented with vancomycin (20 mg/L; Sigma Aldrich) and cycloheximide (200 mg/L; Merck)] to enhance the selection of leuconostocs and inhibit fungi, respectively. Plates were incubated at 30°C for 3 days. For identification, 24 colonies were randomly picked (eight per starter culture mixture) and transferred to liquid MRSv. After overnight incubation at 30°C, cell pellets were obtained by microcentrifugation at 6,000 × *g* for 10 min, which were washed in a Tris-ethylene diaminetetraacetic acid (EDTA)-sucrose buffer [50 mM Tris base (Calbiochem), 1 mM EDTA (Sigma-Aldrich), and 6.7% (m/v) sucrose (Merck); pH 8.0] and subjected to genomic DNA extraction with a Nucleospin 96 tissue kit (Macherey Nagel, Düren, Germany), according to the manufacturer’s instructions. For each isolate, amplicon-based high-throughput sequencing (PacBio) was applied as mentioned above to allow the detection of non-identical copies of the 16S rRNA gene within one isolate.

### 2.3. Meta-metabolomic analysis

#### 2.3.1. Dry mass determinations

The dry mass of the cheese core and rind samples was determined in triplicate with the common oven-drying method. Therefore, approximately 3 g of cheese powder (see below) was dried in an oven (Heraeus, Hanau, Germany) at 105°C for 24 h. All quantifications of substrates and metabolites were expressed on a dry mass basis (m/m).

#### 2.3.2. Cheese powder and extracts

To enable accurate chemical compound extractions, powders of the cheese samples were prepared. Therefore, cheese core and rind samples were frozen using liquid nitrogen (Air Liquide, Paris, France) and subsequently milled into a fine powder with a coffee grinder (De’Longhi KG49, Treviso, Italy). These cheese powders were subjected to two types of extractions. To assess organic acids, free amino acids, and biogenic amines, an aqueous extraction was performed, as described previously (Le Boucher et al., 2016; Zhang et al., 2019). Briefly, 1.0 g of cheese powder was mixed with 9.0 mL of ultrapure water (Milli-Q; Merck) on a rotating wheel at 30 rpm for 30 min at room temperature, followed by centrifugation at 1,000 × *g* for 5 min. Extracts were stored at −25°C until further analysis. To assess volatile organic compounds, ethyl acetate extracts were prepared by mixing 0.5 g of cheese powder with 9.5 mL of ethyl acetate (SupraSolv^®^ grade; Merck) and supplemented with 100 μg/L of toluene-D8 (Sigma-Aldrich) as internal standard (IS). Ethyl acetate extracts were filtered with a Millex Syringe Driven Filter Unit (Millex; Merck) and immediately used for further analysis.

#### 2.3.3. Free amino acid concentration determinations

The concentrations of all 20 proteinogenic amino acids as well as those of 4-aminobutyric acid, citrulline, and ornithine were quantified with external calibration, in triplicate, by ultra-performance liquid chromatography coupled to tandem mass spectrometry (UPLC-MS/MS), using an Acquity system equipped with an HSS T3 column (Waters, Milford, MA, United States), as described previously (Van der Veken et al., 2022). Minor modifications included a constant flow rate of 0.35 mL/min for the mobile phase and the following gradient: 0.0 to 1.0 min, 99% A and 1% B; 1.0 to 8.0 min, 30% A and 70% B; 8.1 min to 10.0 min, 100% B; and 10.1 to 25.0 min, 99% A and 1% B. Samples were prepared by the addition of 300 μL of acetonitrile (Merck) to 100 μL of aqueous extract, followed by microcentrifugation at 18,000 × *g* for 15 min. Then, 900 μL of ultrapure water with 0.10% formic acid and 8.0 mg/L of 2-aminobutyric acid (IS; Sigma-Aldrich) was added to 100 μL of supernatant, and the mixture was filtered with a 0.2-μm LG H-PTFE filter (Millex; Merck) before injection (10 μL) into the column.

#### 2.3.4. Biogenic amine concentration determinations

The concentrations of agmatine, cadaverine, histamine, 2-phenylethylamine, putrescine, spermidine, spermine, tryptamine, and tyramine were quantified with external calibration, in triplicate, by UPLC-MS/MS, using an Acquity system equipped with an HSS T3 column (Waters), as described previously (Van der Veken et al., 2020). Samples were prepared by addition of 300 μL of acetonitrile (Merck) with 0.2% heptafluorobutyric acid (Sigma-Aldrich) to 300 μL of aqueous extracts, followed by microcentrifugation at 18,000 × *g* for 15 min, and filtering with a 0.2-μm LG H-PTFE filter (Millex; Merck) before injection (5 μL) into the column.

#### 2.3.5. Organic acid concentration determinations

The concentrations of citric acid, fumaric acid, gluconic acid, glucuronic acid, hippuric acid, lactic acid, maleic acid, malic acid, malonic acid, orotic acid, oxalic acid, pyruvic acid, succinic acid, and uric acid were quantified with external calibration, in triplicate, by UPLC-MS/MS, using an Acquity system equipped with an HSS T3 column (Waters), as described previously (De Bruyn et al., 2017), except that eluent A contained 2% (v/v) methanol (Merck). Samples were prepared by addition of 700 μL of a 1:1 (v/v) mixture of ultrapure water and methanol (Merck), containing 20 mg/L of salicylic acid (Fluka, Buchs, Switzerland) as IS, to 100 μl of the aqueous extracts, followed by microcentrifugation at 18,000 × *g* for 15 min, and filtering with a 0.2-μm LG H-PTFE filter (Millex; Merck) before injection (2 μL) into the column.

The ratio between D-lactic acid and L-lactic acid was quantified, in duplicate, by UPLC-MS/MS, using an Acquity system equipped with an Astec Chirobiotic column (Supelco; Bellefonte, PA, United States). The mobile phase consisted of an isocratic flow of 15% ultrapure water with 33.3 mM ammonium acetate (VWR International) and 85% acetonitrile (Merck) at a constant flow rate of 0.6 mL/min. Samples were prepared by addition of 100 μL of aqueous extract to 900 μL of a solution containing 85% acetonitrile (Merck), 15% ultrapure water, 0.386 g/L of ammonium acetate (VWR International), and 4.0 mg/L of salicylic acid (IS; Fluka). The mixtures were vortexed for 5 min, followed by microcentrifugation at 18,000 × *g* for 15 min, and filtering with a 0.2-μm LG H-PTFE filter (Millex; Merck) before injection (10 μL) into the column.

#### 2.3.6. Volatile organic compound concentration determinations

The concentrations of targeted volatile organic compounds present in ethyl acetate extracts were quantified with external standards by liquid injection gas chromatography with triple-quad tandem mass spectrometry (LI-GC-TQ-MS/MS), using a Trace 1,300 gas chromatograph equipped with a Dbwax-etr column (Thermo Fisher Scientific) and coupled to a TSQ 8000 EVO triple quadrupole mass spectrometer (Interscience, Breda, Netherlands), as described previously (Díaz-Muñoz et al., 2021).

### 2.4. Sensory analysis

Cheeses were organoleptically assessed by a company panel experienced in cheese tasting. This panel consisted of 10 people spread over sex and age. After 36, 45, 75, and 100 weeks of ripening, the panel members scored each Gouda cheese from each production batch individually on a scale from 0 to 20, whereafter Z-scores were calculated for all cheeses assessed by one person, using the formula Z-score = (x – μ)/σ, with μ the average and σ the standard deviation of all scores given by that person. Per cheese, the average Z-score of all panel members was then calculated and used as sensory quality criterium.

### 2.5. Statistical analysis

All statistical analyses were performed in R (version 4.1.0; R Core Team, 2021). One-way analysis of variance (ANOVA) was performed to compare differences in relative abundances of bacteria between starter culture rotations and ripening times, followed by *post-hoc* pairwise comparisons with Tukey’s test. A principal component analysis (PCA) was performed to compare species compositions of all cheese samples at all time points. A scaled PCA (Z-score transformation) was performed to compare *Lactococcus* ASVs at 36 weeks of ripening among batches and to compare all metabolites at 36, 45, 75, and 100 weeks of ripening, in each case between cores and rinds. For correlation analysis, only species with an average relative abundance of ≥0.2% across all cheese cores after 36, 45, 75, and 100 weeks of ripening were considered. Spearman correlation coefficients were calculated when data from different ripening times were used, whereas Pearson correlation coefficients were calculated for correlations with the sensory score at one ripening time. Spearman correlations between species and metabolites were visualized in a heatmap using the ComplexHeatmap package (version 2.0.0; Gu et al., 2016). Hierarchical clustering analysis was based on the Ward’s method (clustering method set to “Ward.D2”). For all statistical tests, results with a *p*-value < 0.05 were considered significantly different.

Microbial intra-sample diversity (alpha diversity) was assessed by calculating the inverse Simpson diversity index, applying the vegan package (version 2.5–7; Oksanen et al., 2020). Microbial inter-sample diversity (beta diversity) was assessed by conducting a permutational multivariate analysis of variance (PERMANOVA), based on Bray–Curtis dissimilarity scores, applying the RVAideMemoire package (version 0.9–80; Hervé, 2021).

## 3. Results

### 3.1. Metagenetics of cheese cores and rinds

Cores and rinds of Gouda cheeses from 23 cheese production batches, with sensory scores L, G or E, produced in the same dairy factory with starter culture mixture A, B or C, were investigated after 36, 45, 75, and 100 weeks of ripening through metagenetics (for the rinds only after 36 weeks of ripening) and meta-metabolomics. Nine cheese batches (score G) were also assessed after 26 and 31 weeks of ripening.

#### 3.1.1. Bacterial species-level metagenetics

**Figure 2:**
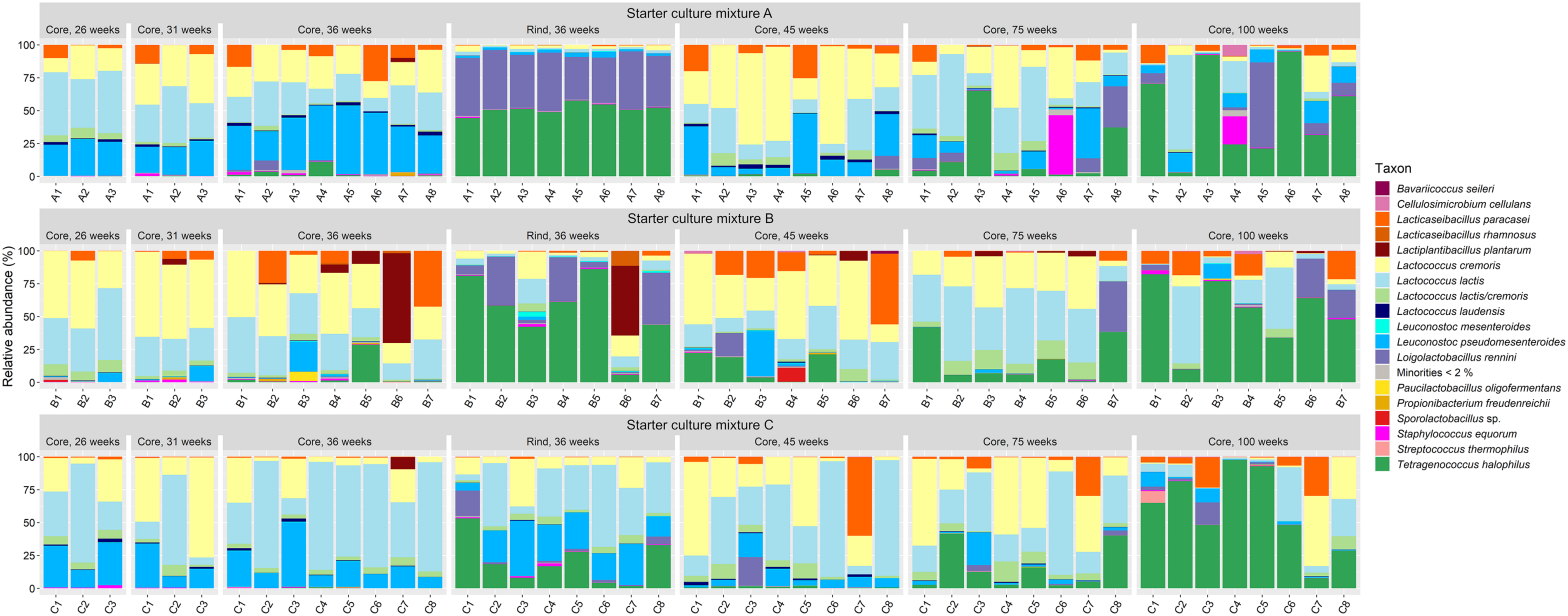
Bacterial species dynamics, expressed as relative abundances based on amplicon sequence variants of the full-length 16S rRNA gene, for cheese cores after 26, 31, 36, 45, 75, and 100 weeks of ripening and rinds after 36 weeks of ripening. The Gouda cheeses were made with three different mixed-strain starter cultures (A, B, and C). The numbers indicate the cheeses of the 23 different batch productions.

In general, the bacterial species diversity in the cheese cores was rather small for all LAB starter culture mixtures used throughout the ripening period (Figure 2). The most abundant bacterial species were *Lc. lactis* and *Lc. cremoris*. *Leuconostoc pseudomesenteroides* was the third most abundant bacterial species up to 45 weeks of ripening. The most abundant NSLAB species were *Lacc. paracasei*, which had its maximum relative abundance after 45 weeks of ripening, and *Tetragenococcus halophilus*, which was the most abundant NSLAB species after 100 weeks of ripening. *Lactiplantibacillus plantarum*, *Loigolactobacillus rennini*, and *Staphylococcus equorum* were always found at more than 15% of relative abundance in at least one cheese batch, whereas *Lactococcus laudensis* was found in almost all cheese batches, but always at a low relative abundance.

The cores of the Gouda C cheeses contained significantly more *Lc. lactis* compared to those of the other cheeses, whereas those of the Gouda B cheeses contained significantly more *Lc. cremoris* than those of the Gouda C cheeses after 36 weeks of ripening (Figure 2). After 26 and 36 weeks of ripening, the relative abundance of *Leuc. pseudomesenteroides* was significantly lower in the cores of the Gouda B cheeses than in those of the other ones. Only in one batch of the Gouda B cheeses (B3), *Leuc. pseudomesenteroides* was of high relative abundance throughout the ripening period. After 36 weeks of ripening, the Gouda A cheeses contained significantly more *Leuc. pseudomesenteroides* compared to the Gouda C cheeses, whereas the Gouda B cheeses contained significantly more NSLAB species than the Gouda C ones. Throughout the ripening period, *Lc. laudensis* was found in 24% of the cheese cores at a relative abundance of ≥1.0%, but after 36, 45, and 75 weeks of ripening, its relative abundance was significantly lower in the Gouda B cheeses compared to the Gouda A cheeses, and also significantly lower after 75 weeks of ripening compared to the Gouda C ones.

The cheese rinds (36 weeks of ripening) displayed a significantly lower relative abundance of *Lc. lactis* and *Lc. cremoris* compared to the cheese cores for all starter culture mixtures (Figure 2). Further, the bacterial diversity of the rinds of the Gouda A and B cheeses was different from those of the Gouda C cheeses. The Gouda A and B cheeses had a significantly higher relative abundance of *Loil. rennini* and *T. halophilus* in the rinds compared to the cores. Furthermore, the Gouda A cheeses contained less *Lc. laudensis* and *Leuc. pseudomesenteroides* in the rinds compared to the cores, whereas the Gouda B cheeses had more *Leuc. mesenteroides* in the rinds compared to the cores. Among the rinds, the Gouda C cheeses had more *Lc. lactis* and *Leuc. pseudomesenteriodes* and less *T. halophilus* compared to those of the other ones. The Gouda B rinds contained significantly more *Leuc. mesenteroides* compared to the other ones. All Gouda cheese rinds had significantly different levels of *Loil. rennini*, with the Gouda A rinds representing the highest and the Gouda C rinds the lowest relative abundances.

The alpha diversity of the bacterial species compositions displayed a bell shape as a function of the ripening time for the Gouda A and B cheeses, reaching its maximum at 36 and 45 weeks of ripening, respectively, whereas it was almost constant for the Gouda C cheeses (Supplementary Figure S1). This diversity index only differed significantly between cheeses made with different starter culture mixtures after 36 weeks of ripening in that the alpha diversity of the Gouda C cheeses was significantly lower in the cores and higher in the rinds compared to those of the other cheeses. The generally lower alpha diversity for the Gouda C cheeses compared to the other ones reflected a lower relative abundance of NSLAB species. The beta diversity of the bacterial species compositions among the Gouda cheeses roughly followed an upside down bell curve, with the highest similarities after 26 and 31 weeks of ripening, especially among the Gouda A and C cheeses, and after 75 and 100 weeks of ripening for all cheeses (Supplementary Figure S1). After 36 weeks of ripening, the Gouda A, B, and C cheeses had a significantly different bacterial species composition, for both the cores and the rinds when compared to each other. The bacterial species compositions were also significantly different between cores and rinds among the Gouda A and B cheeses.

A PCA indicated that *Lc. lactis*, *Lc. cremoris*, *T. halophilus*, *Leuc. pseudomesenteroides*, *Lacc. paracasei*, and *Loil. rennini* explained most of the species variance between the samples (Supplementary Figure S2). Most of the rinds of the 36-week Gouda A and B cheeses clustered with those of the cheeses of 100 weeks of ripening, based on the high relative abundances of *T. halophilus* and *Loil. rennini*. The cores of Gouda cheeses of 26, 31, 36, and 45 weeks of ripening were spread over three clusters, namely a cluster characterized by a high relative abundance of *Lc. cremoris*, a cluster of mainly Gouda C cheeses characterized by a high relative abundance of *Lc. lactis*, and a cluster of mainly cheeses ripened for 36 weeks and characterized by high relative abundances of *Leuc. pseudomesenteroides*, *Lc. lactis*, and *Lc. cremoris*.

Spearman correlation coefficients showed that *Lc. lactis* and *Lc. cremoris* were negatively correlated with *T. halophilus* and *Loil. rennini* (Supplementary Figure S3). The two latter species were positively correlated. *Lactococcus laudensis* was positively correlated with *Lc. cremoris*, *Lc. lactis*, and *Leuc. pseudomesenteroides*. Further, *Lacp. plantarum, Lacc. rhamnosus*, and *Propionibacterium freudenreichii* were positively correlated.

#### 3.1.2. Bacterial ASV-level metagenetics

To allow insights below species level, the compositions of the ASVs obtained were assessed for all starter LAB and the most abundant NSLAB species.

##### 3.1.2.1. Lactococcus

In the case of the genus *Lactococcus*, for all cheese cores and rinds, 21 ASVs belonged to *Lc. cremoris*, 6 ASVs to *Lc. lactis*, and 15 ASVs were considered as intermediate, as they displayed an identity varying between 99.39 and 99.73% with both type strains.

**Figure 3:**
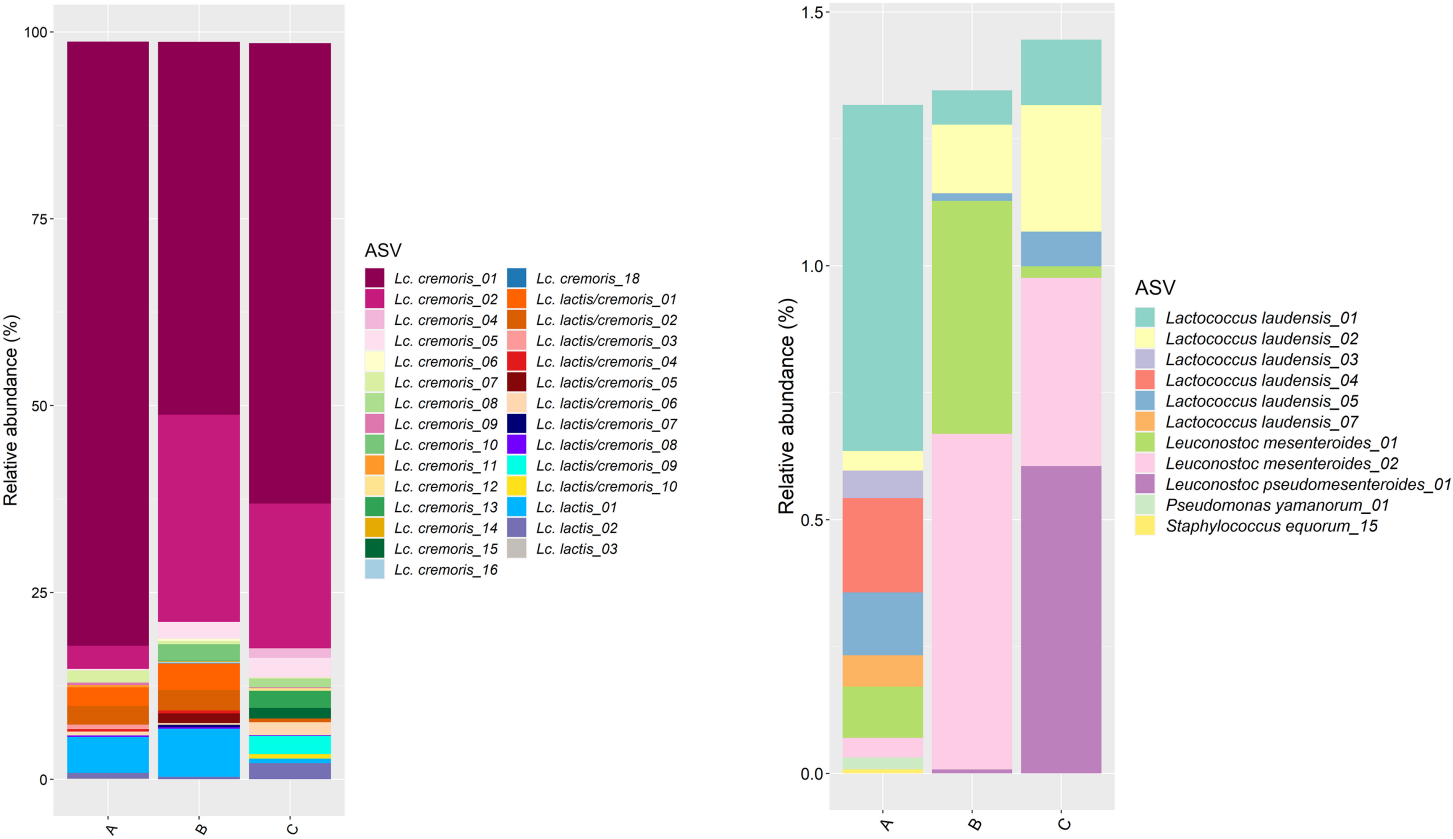
Taxonomic assessment on amplicon sequence variant (ASV) level of the bacterial full-length 16S rRNA gene for the three commercial starter culture mixtures (A, B, and C) investigated. Left, ASVs of *Lactococcus cremoris* and *Lactococcus lactis* (ASVs belonging to *Lc. cremoris* or *Lc. lactis* that could not be assigned to one of those species are indicated as *Lc. lactis/cremoris*). Right, ASVs of all other bacterial species identified in the starter culture mixtures.

Concerning the starter culture mixtures, more than 80% of the ASVs belonged to *Lc. cremoris*, with the *Lc. cremoris*_01 ASV as the most abundant one, followed by the *Lc. cremoris*_02 ASV (Figure 3). The ASVs of *Lc. lactis* summed up to 5.7, 6.8, and 2.8% in starter culture mixture A, B, and C, respectively. The ASVs of *Lc. laudensis* were present in all starter culture mixtures at a relative abundance comparable to that of the leuconostocs (see below).

**Figure 4:**
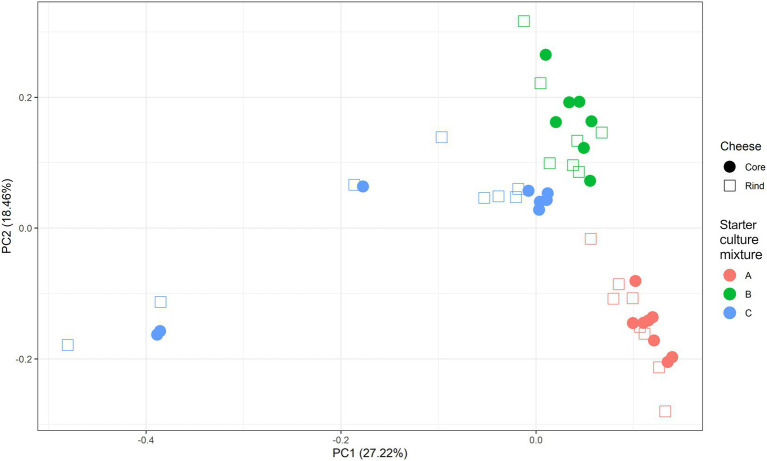
Scaled principal component analysis of all amplicon sequence variants based on the full-length 16S rRNA gene of *Lactococcus cremoris* and *Lactococcus lactis* identified in the cores (full symbols) and rinds (open symbols) from 23 different Gouda cheese batch productions made with three different mixed-strain starter cultures (A, red; B, green; C, blue) after 36 weeks of ripening.

A scaled PCA of all ASVs separated the 23 cheese production batches at 36 weeks of ripening based on starter culture mixture used; the ASVs corresponding with the cores and rinds showed no differences (Figure 4). The Gouda C cheeses had significantly less of the *Lc. cremoris*_01 ASV and more of the *Lc. lactis*_02 ASV after 36 weeks (both cores and rinds) and 45 weeks (cores) of ripening compared to the other cheeses (Supplementary Figure S4). Furthermore, the Gouda A cheeses had significantly less of the *Lc. cremoris*_02 ASV in the rinds than in those of the other cheeses after 36 weeks of ripening, whereas the Gouda C cheeses had then more of the *Lc. cremoris*_04 ASV in both cores and rinds than in those of the other cheeses. The Gouda B cheeses had more of the *Lc. cremoris*_06 ASV after 36 (both cores and rinds), 45 (cores), and 75 (cores) weeks of ripening than in those of the other cheeses. After 100 weeks of ripening, no significant differences between the Gouda cheeses was found. For the Gouda A cheeses, the relative abundance of the *Lc. cremoris*_01 ASV within the *Lactococcus* genus decreased as a function of the ripening time and was significantly lower after 75 weeks of ripening compared to that after 36 and 45 weeks of ripening and significantly lower after 100 weeks of ripening compared to 45 weeks. Among the Gouda B cheeses, the *Lc. lactis*_01 ASV increased as a function of the ripening time, which was significantly higher between 36 and 45 weeks of ripening compared to 100 weeks and after 45 weeks of ripening compared to 75 weeks. In contrast, the *Lc. cremoris*_01 ASV decreased as a function of the ripening time, and was significantly lower after 75 and 100 weeks of ripening compared to 36 and 45 weeks. In addition, the *Lc. cremoris*_02 ASV was significantly lower after 100 weeks of ripening compared to 45 weeks. Concerning the Gouda C cheeses, the relative abundances of the ASVs corresponding with *Lc. cremoris* and *Lc. lactis* were not significantly different during ripening.

##### 3.1.2.2. Leuconostoc

The Gouda C cheeses mainly contained the *Leuc. pseudomesenteroides*_01 ASV (Supplementary Figure S4). This ASV was also the most abundant *Leuconostoc* ASV in the Gouda A and B cheeses. The *Leuc. pseudomesenteroides*_02 ASV was found in all Gouda A cheeses (but not at all ripening times), mostly in a ratio of 1:3 compared to the *Leuc. pseudomesenteroides*_01 ASV, whereas it appeared more randomly in all Gouda B cheeses (not at all ripening times) and in two Gouda C cheeses. The *Leuc. pseudomesenteroides*_03 ASV appeared at later time points of the ripening stage in most Gouda C cheeses, as well as in three Gouda A cheeses and at all ripening times in one Gouda B cheese.

All *Leuc. mesenteroides* ASVs were only found in the Gouda cheeses when the *Leuconostoc* genus was present at a low relative abundance (Supplementary Figure S4). This was also the case in the pure starter culture mixtures, displaying a relative abundance of 0.14, 1.13, and 1.00% of leuconostocs in starter culture mixture A, B and C, respectively. Moreover, the *Leuc. pseudomesenteroides*_02 and *Leuc. pseudomesenteroides*_03 ASVs were not found in the starter culture mixtures, despite their presence in the cheeses. Metagenetic analysis of the starter culture mixtures allowed to detect non-identical copies of the 16S rRNA gene within one isolate (50% of the isolates, Supplementary Figure S5). Indeed, 12 isolates from starter culture mixtures A and B contained both the *Leuc. pseudomesenteroides*_01 and *Leuc. pseudomesenteroides*_02 ASVs, in a ratio of 3:1, which was in accordance with a copy number of four for the 16S rRNA gene of *Leuc. pseudomesenteroides* (Stoddard et al., 2015). The other isolates each contained only one ASV, indicating that the four copies were identical. Hence, three different ASV clusters of *Leuc. pseudomesenteroides* were present. A first cluster (ASV cluster 1) contained only the *Leuc. pseudomesenteroides*_01 ASV, as found in the Gouda C cheeses. A second cluster (ASV cluster 2) contained the *Leuc. pseudomesenteroides*_01 and *Leuc. pseudomesenteroides*_02 ASVs in a 3:1 ratio, as found in the Gouda A cheeses. ASV cluster 3 represented the *Leuc. pseudomesenteroides*_03 ASV, which was spread among the Gouda A, B, and C cheeses.

Differences between ASVs present in the cores and rinds after 36 weeks of ripening were small for the Gouda C cheeses (Supplementary Figure S4). In the rinds of the Gouda A cheeses, the *Leuc. mesenteroides*_01 and *Leuc. mesenteroides*_02 ASVs appeared, whereas the rinds of the Gouda B cheeses had significantly more of the *Leuc. mesenteroides*_02 ASV and less of the *Leuc. pseudomesenteroides*_01 and *Leuc. pseudomesenteroides*_02 ASVs.

##### 3.1.2.3. *Lacticaseibacillus* and *Lactiplantibacillus*

A total of 67 *Lacc. paracasei*, 22 *Lacp. plantarum*, eight *Lacc. rhamnosus*, and one *Lacc. casei* ASVs were found across all Gouda cheese cores and rinds, albeit that most were only found at low relative abundance (Supplementary Figure S6). The appearance of different *Lacticaseibacillus* and *Lactiplantibacillus* ASVs was not linked with the ripening time, the cheese cores or rinds, or the LAB starter culture mixtures applied, but these ASVs were production batch-dependent. In most cheese production batches, the same ASVs were found at different ripening times.

##### 3.1.2.4. *Tetragenococcus halophilus* and *Loigolactobacillus rennini*

**Figure 5:**
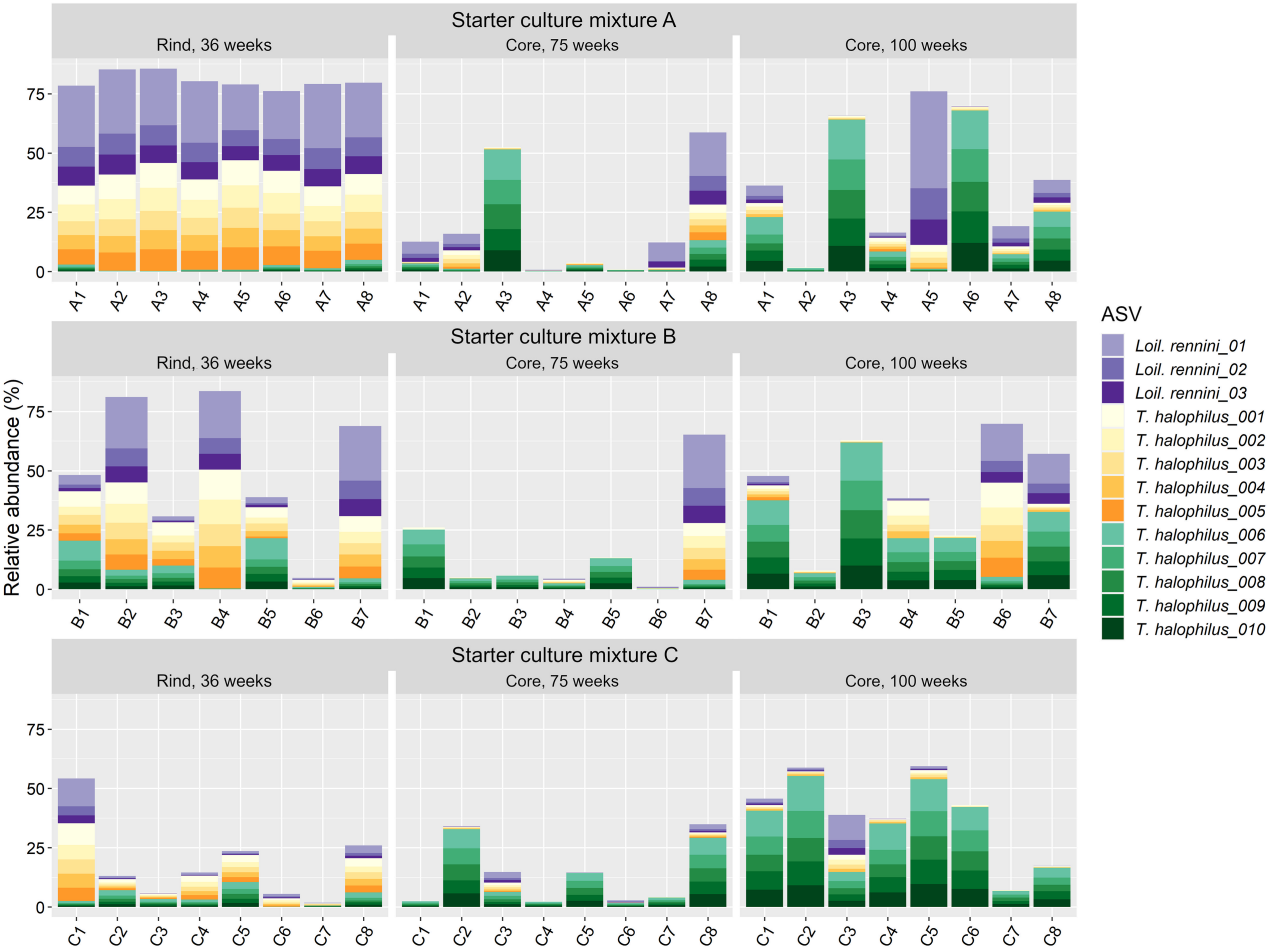
Relative abundance of the amplicon sequence variants (ASVs) based on the full-length 16S rRNA gene of the non-starter lactic acid bacteria (NSLAB) species *Loigolactobacillus rennini* (three most abundant ones) and *Tetragenococcus halophilus* (10 most abundant ones) identified in the Gouda cheeses from 23 different batch productions made with three different mixed-strain starter cultures (A, B, and C) after 36 weeks of ripening (rinds) and 75 and 100 weeks of ripening (cores). The numbers indicate the cheeses of the 23 different batch productions. The ASVs *T. halophilus*_001–005 form ASV cluster 1, whereas the ASVs *T. halophilus_*006–010 form ASV cluster 2.

For *T. halophilus*, 108 different ASVs were found, which was the highest number of ASVs for any LAB species in the Gouda cheeses of the present study. The 10 most abundant *T. halophilus* ASVs could be grouped in two clusters of five highly correlated ASVs, found in equal ratios among the cheese production batches, namely ASV cluster 1 (ASVs *T. halophilus_*001–005) and ASV cluster 2 (ASVs *T. halophilus*_006–010) (Figure 5). *Loigolactobacillus rennini* had nine different ASVs, of which only three were highly abundant. They were found in a constant ratio of 3:1:1, suggesting one main ASV cluster.

#### 3.1.3. Fungal genus-level metagenetics

**Figure 6:**
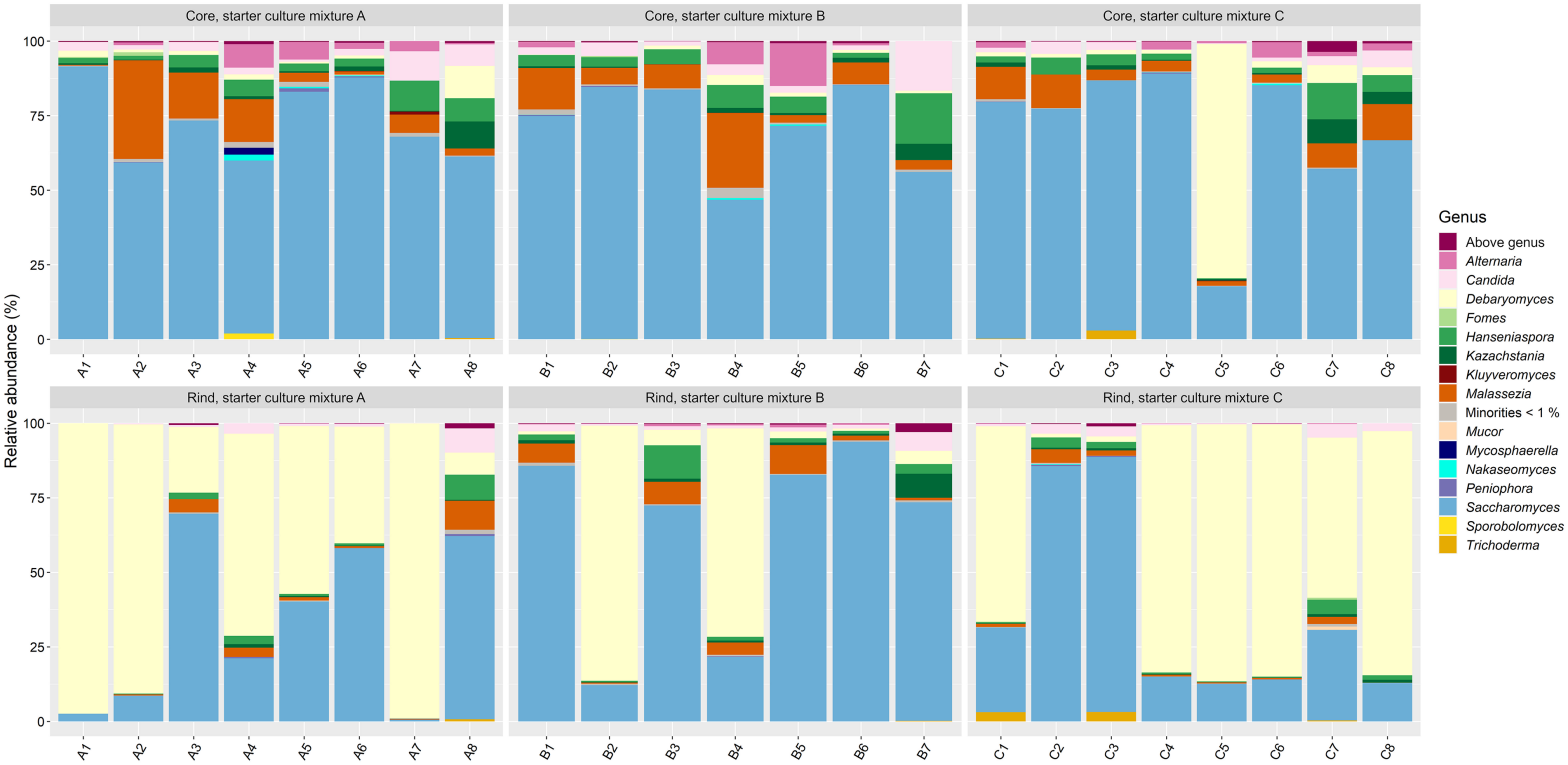
Taxonomic assessment on fungal genus level, expressed as relative abundances based on amplicon sequence variants (ASVs) of the internal transcribed spacer (ITS) region ITS1 of the rRNA transcribed unit, for cheese cores and rinds after 36 weeks of ripening. The Gouda cheeses were made with three different mixed-strain starter cultures (A, B, and C). The numbers indicate the cheeses of the 23 different batch productions.

As fungi were not expected to be abundant in the Gouda cheeses and since 36 weeks of ripening was considered as the most interesting time point regarding the bacterial species diversity, the fungal species diversity was only assessed at this ripening time. The main genera present were *Debaryomyces* and *Saccharomyces* (Figure 6). Both genera accounted for >75.0% of the relative abundances in 75% of all cheese production batches. Five other genera were present at more than 5.0% of relative abundance in at least one cheese batch, namely *Alternaria*, *Candida*, *Hanseniaspora*, *Kazachstania*, and *Malassezia*.

The fungal alpha diversity was not significantly different between the Gouda A, B, and C cheeses for both cores and rinds, and was not significantly different between the cores and rinds among the Gouda cheeses made with the same starter culture mixture. The beta diversity of the fungal genera compositions between the cores and rinds was only significantly different for the Gouda A cheeses. For all cheese production batches, the relative abundance of the genus *Saccharomyces* was significantly higher in the cores compared to the rinds (average relative abundances of 71.3 and 43.0%, respectively), whereas the relative abundance of the genus *Debaryomyces* was significantly lower in the cores compared to the rinds (average relative abundances of 5.3 and 48.1%, respectively).

### 3.2. Meta-metabolomics of cheese cores and rinds

#### 3.2.1. Free amino acids

**Figure 7:**
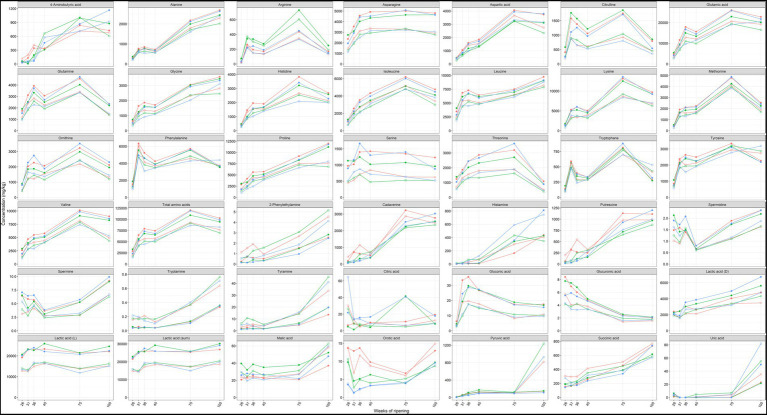
Average concentrations, expressed in mg/kg dry mass, of amino acids, biogenic amines, and organic acids in the cores (full symbols) and rinds (open symbols) of Gouda cheeses made with three different mixed-strain starter cultures (A, red; B, green; C, blue) as a function of the ripening time. The concentrations of cysteine, maleic acid, malonic acid, hippuric acid, and fumaric acid were below the limit of determination, whereas agmatine was not found.

Amino acids were the most abundant metabolites found in the Gouda cheese cores and rinds, with concentrations of total free amino acids ranging from 15.1 to 120.2 g/kg (Figure 7). Glutamic acid, lysine, leucine, proline, and valine were, in decreasing concentrations, the most abundant free amino acids among all cheese production batches and ripening times.

The concentrations of all amino acids mentioned above displayed an increasing trend from 26 up to 75 weeks of ripening, both in the cheese cores and rinds, with a stagnation or small decrease between 36 and 45 weeks of ripening, followed by a major increase between 45 and 75 weeks. The concentrations were in general higher after 100 weeks of ripening than after 45 weeks but lower than after 75 weeks. The concentrations of most amino acids were significantly higher in the cheese cores compared to the rinds at all ripening times.

The concentrations of arginine were among all samples 88% higher in the Gouda B cheeses compared to the other ones, which was significant for the cheese rinds after 36 weeks of ripening and for all cheese cores and rinds after 45, 75, and 100 weeks of ripening. The concentrations of most other amino acids were lower in the Gouda B cheeses compared to the other ones.

#### 3.2.2. Biogenic amines

The concentrations of cadaverine, histamine, 2-phenylethylamine, putrescine, tryptamine, and tyramine increased significantly in the Gouda cheese cores and rinds from 45 to 100 weeks of ripening, with the main increase between 45 and 75 weeks (Figure 7). In the case of cadaverine, histamine, and putrescine, concentrations around 1 g/kg were reached after 75 and 100 weeks of ripening. The concentrations of 2-phenylethylamine, tryptamine, and tyramine were significantly higher in the cheese rinds compared to the cores at all ripening times. A significantly higher concentration in the rind was also the case for histamine after 31, 36, 45, and 75 weeks of ripening and for putrescine and cadaverine after 36 and 45 weeks of ripening. The low concentrations of spermine and spermidine were significantly higher in the cheese cores at all ripening times, except for spermidine after 36 and 45 weeks of ripening.

The concentrations of cadaverine and putrescine were significantly higher in the Gouda A cheeses compared to the other ones, after 31 weeks of ripening in the cores and after 36 weeks in the cores and rinds.

#### 3.2.3. Organic acids

Lactic acid was the main organic acid present in the Gouda cheeses, with an average concentration of 25.8 g/kg in the cores and 17.2 g/kg in the rinds (Figure 7). The concentrations of D-lactic acid and succinic acid increased between 45, 75, and 100 weeks of ripening in both the cores and rinds. The low concentrations of citric acid were more than twice as high in the cores of the Gouda C cheeses compared to those of the other ones after 26 and 36 weeks of ripening. Concerning the cheese rinds, the concentrations of pyruvic acid were significantly lower for the Gouda C cheeses compared to the other ones after 36 weeks of ripening. However, pyruvic acid only significantly increased in the rinds of all Gouda cheeses, with a ninefold increase between 75 and 100 weeks of ripening. Malic acid had higher concentrations in the cores of the Gouda B cheeses, which was significant after 26, 36, and 45 weeks of ripening. The Gouda A cheeses had higher concentrations of orotic acid, which was significant in the cores after 31 and 36 weeks of ripening and in the rinds after 36 and 45 weeks.

#### 3.2.4. Volatile organic compounds

**Figure 8:**
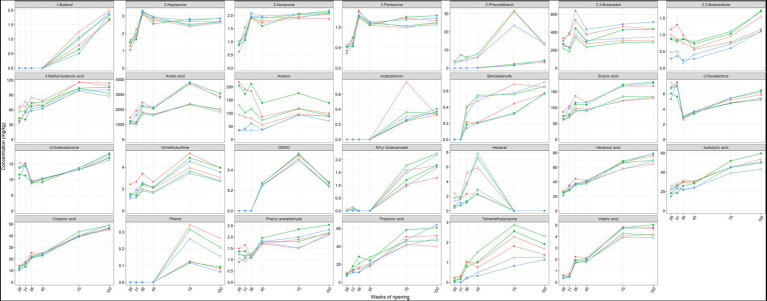
Average concentrations, expressed in mg/kg dry mass, of volatile organic compounds in the cores (full symbols) and rinds (open symbols) of Gouda cheeses made with three different mixed-strain starter cultures (A, red; B, green; C, blue) as a function of the ripening time.

Acetic acid, acetoin, and 2,3-butanediol were the main volatile organic compounds found in the Gouda cheeses examined (Figure 8). The concentrations of these compounds were significantly higher in the cores than in the rinds at all ripening times. In contrast, the concentrations of benzaldehyde, δ-decalactone, ethyl dodecanoate, hexanal, octanoic acid, 2-pentanone, 2-phenylethanol, and trimethylpyrazine were significantly higher in the rinds for at least three ripening times. The concentrations of benzaldehyde, dimethylsulfone, δ-dodecalactone, phenyl acetaldehyde, 2-phenylethanol, tetramethylpyrazine, and all short-chain fatty acids increased as a function of the ripening time, mainly between 45 and 75 weeks.

The concentrations of acetoin were lower in the Gouda C cheeses, in both cores and rinds, compared to the other ones at all ripening times. This was threefold lower in the cores after 26 and 31 weeks of ripening and in both the cores and rinds after 36 weeks. In general, the Gouda B cheeses had the highest acetoin concentrations. A similar trend was found for the concentrations of 2,3-butanedione. In contrast, the concentrations of 2,3-butanediol were higher in the Gouda C cheeses compared to the other ones, which was significant in the cores and rinds after 36 weeks of ripening. The concentrations of dimethyl sulfone were significantly higher in the cores of the Gouda A cheeses compared to those of the other ones after 26, 31, and 36 weeks of ripening.

### 3.3. Impact of the starter culture mixture and species

**Figure 9:**
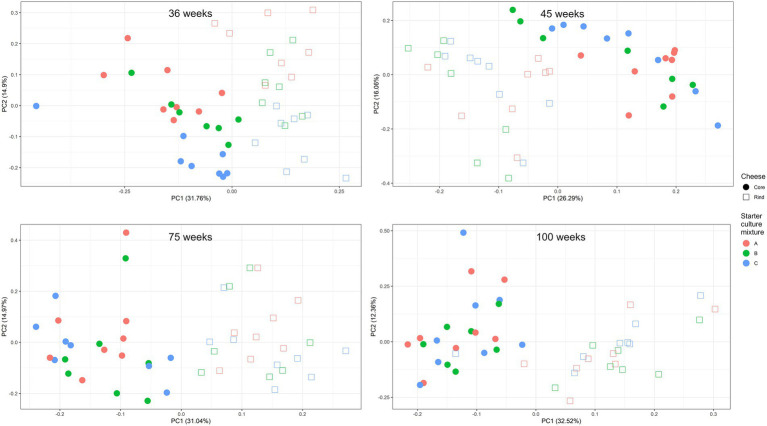
Scaled principal component analysis of the concentrations of the non-volatile and volatile organic compounds of Figures 6, 7, measured in the cores (full symbols) and rinds (open symbols) of Gouda cheeses made with three different mixed-strain starter cultures (A, red; B, green; C, blue), according to the ripening time.

After 36 weeks of ripening, a PCA on the scaled values of all metabolites clustered the cheese production batches mainly according to the starter culture mixture used (Figure 9). The effect of the starter culture mixture decreased during ripening. At all ripening times, a clear distinction was found between cheese cores and rinds.

**Figure 10:**
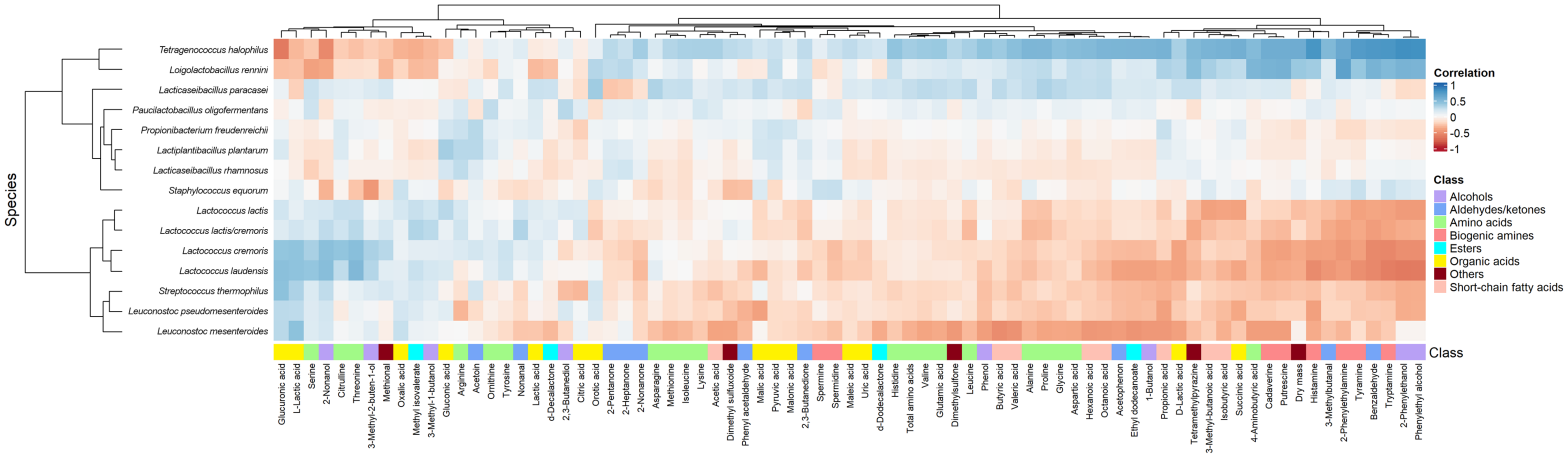
Spearman correlations between the relative abundances of the major bacterial species and the metabolite concentrations found in the Gouda cheeses from 23 different batch productions at all ripening times. Metabolites without any significant correlation are not shown.

According to the clustered heatmap of Spearman correlations, two main clusters could be distinguished that correlated the metabolites and main species throughout ripening (Figure 10). One cluster contained all *Lactococcus* and *Leuconostoc* species as well as *S. thermophilus*, whereas the other cluster contained *P. freudenreichii*, *St. equorum*, and the most abundant NSLAB. All species of the former cluster were positively correlated with L-lactic acid, whereas the species *Lacc. paracasei*, *T. halophilus*, and *Loil. rennini* were positively correlated with D-lactic acid. Most amino acids were positively correlated with *Lacc. paracasei* and *T. halophilus*. Serine, threonine, and citrulline were positively correlated with *Lactococcus*. All biogenic amines, except for spermine and spermidine, were positively correlated with *Loil. rennini* and *T. halophilus*. These LAB species were also positively correlated with dry mass, reflecting their osmotolerance. *Tetragenococcus halophilus* was positively correlated with short-chain fatty acids and, in general, with most metabolites that increased during ripening.

Intra-species differences were also found. Acetoin and its related metabolites, 2,3-butanediol and 2,3-butanedione, and citric acid had different correlations among ASVs of *Lc. lactis*, *Lc. cremoris* and *Leuc. pseudomesenteroides* (Table 1), whereas the two main *T. halophilus* ASV clusters had different correlations with specific amino acids, biogenic amines, and other aroma compounds after 75 weeks of ripening (Table 2). Since it was hypothesized that the ASV clusters of *Leuc. pseudomesenteroides* did not straightforwardly correspond with the three ASVs identified, correlations with the *Leuc. pseudomesenteroides*_01 ASV differed from those with ASV cluster 1. The differences were most pronounced when only the cheese cores of 36 weeks of ripening were considered. ASV cluster 1 and ASV cluster 3 were negatively correlated with the total amino acid concentration and acetoin, but positively with lactic acid, whereas the opposite was the case for ASV cluster 2, suggesting that the latter was more related to flavor formation, whereas the former clusters participated more in acidification (Table 3). This was also found for the higher concentrations of most amino acids at 36 weeks of ripening for the Gouda A cheeses containing ASV cluster 2.

**Table 1:** Spearman correlations between acetoin concentrations and other pyruvate metabolites and a selection of significantly different amplicon sequence variants (ASVs) of the starter culture mixtures applied.

ASV / ASV clusters	Compounds			
	2,3-Butanediol	2,3-Butanedione	Acetoin	Citric acid
*Lc. cremoris_*01	**−0.27**	−0.04	**0.33**	**−0.18**
*Lc. cremoris_*02	−0.01	**−0.41**	−0.17	0.17
*Lc. cremoris_*04	**0.36**	**−0.63**	**−0.69**	**0.59**
*Lc. cremoris_*06	**−0.38**	**0.30**	**0.59**	**−0.32**
*Lc. lactis_*01	0.15	**−0.18**	0.12	0.09
*Lc. lactis_*02	**0.21**	**−0.59**	**−0.42**	**0.28**
*Leuc. pseudomesenteroides_*01	**0.18**	**−0.25**	**−0.23**	0.06
*Leuc. pseudomesenteroides* ASV cluster 1	**0.29**	**−0.49**	**−0.46**	**0.26**
*Leuc. pseudomesenteroides* ASV cluster 2	−0.15	**0.42**	**0.45**	**−0.47**
*Leuc. pseudomesenteroides* ASV cluster 3	**0.30**	**−0.62**	**−0.53**	**0.41**

Significant correlations are represented in bold. The *Leuc. pseudomesenteroides* ASV cluster 1 equals the relative abundance of the *Leuc. pseudomesenteroides*_01 ASV minus three times the relative abundance of the *Leuc. pseudomesenteroides*_02 ASV.

**Table 2:** Spearman correlations between compounds and the two main *Tetragenococcus halophilus* ASV clusters and *Loigolactobacillus rennini* after 75 weeks of ripening.

Compound	*T. halophilus* ASV cluster 1	*T. halophilus* ASV cluster 2	*Loil. rennini*	Class
Phenyl acetaldehyde	**−0.44**	0.09	**−0.62**	Aldehyde
4-Aminobutyric acid	**0.60**	−0.07	**0.73**	Amino acid
Arginine	**−0.51**	−0.21	**−0.52**	Amino acid
Aspartic acid	0.38	0.04	0.41	Amino acid
Citrulline	−0.19	**−0.45**	−0.25	Amino acid
Histidine	0.32	−0.14	0.28	Amino acid
Lysine	**−0.53**	0.06	**−0.65**	Amino acid
Ornithine	**−0.54**	0.24	**−0.68**	Amino acid
Phenylalanine	−0.22	0.07	−0.34	Amino acid
Proline	0.38	0.10	0.38	Amino acid
Serine	**−0.43**	−0.03	**−0.54**	Amino acid
Threonine	**−0.43**	−0.19	**−0.42**	Amino acid
Tryptophane	−0.28	0.13	−0.29	Amino acid
Tyrosine	−0.36	−0.15	−0.37	Amino acid
2-Phenylethylamine	**0.65**	−0.19	**0.74**	Biogenic amine
Cadaverine	**0.65**	−0.13	**0.82**	Biogenic amine
Histamine	−0.14	**0.71**	−0.25	Biogenic amine
Putrescine	**0.61**	−0.05	**0.77**	Biogenic amine
Tryptamine	0.36	−0.07	0.39	Biogenic amine
Tyramine	−0.20	0.20	−0.24	Biogenic amine
D-Lactic acid	0.23	−0.24	**0.57**	Organic acid
Acetic acid	−0.10	−0.18	0.02	Short-chain fatty acid
Butyric acid	0.29	0.17	0.35	Short-chain fatty acid
Hexanoic acid	0.22	−0.06	0.31	Short-chain fatty acid
Isobutyric acid	0.15	−0.18	0.13	Short-chain fatty acid
Octanoic acid	0.18	−0.02	0.19	Short-chain fatty acid
Propionic acid	**0.47**	0.15	**0.57**	Short-chain fatty acid
Valeric acid	0.37	−0.07	**0.45**	Short-chain fatty acid
Dimethylsulfone	**0.48**	**−0.46**	**0.59**	Sulfur compound
Methional	−0.30	−0.04	−0.36	Sulfur compound
pH	**0.65**	0.11	**0.73**	
Dry mass	0.20	**0.45**	0.14	

Significant correlations are represented in bold.

**Table 3:** Spearman correlations between some metabolite concentrations and *Leuconostoc pseudomesenteroides* amplicon sequence variants (ASVs) and ASV clusters, for Gouda cheese cores after 36 weeks of ripening.

Compound	*Leuc. p.* _01	ASV cluster 1	ASV cluster 2	ASV cluster 3
Total amino acids	0.35	−0.19	0.34	−0.14
Lactic acid	−0.01	0.28	−0.34	0.28
2,3-Butanediol	0.41	**0.57**	−0.20	**0.54**
2,3-Butanedione	0.09	**−0.52**	**0.57**	**−0.65**
Acetoin	−0.20	**−0.69**	0.41	**−0.54**
Citric acid	0.04	**0.43**	**−0.57**	**0.42**

Significant correlations are represented in bold. *Leuc. p._*01 indicates the *Leuc. pseudomesenteroides*_01 ASV. ASV clusters 2 and 3 resulted in the same values as the *Leuc. pseudomesenteroides*_02 ASV and the *Leuc. pseudomesenteroides*_03 ASV, respectively.

### 3.4. Sensory analysis

The initial classification of the Gouda cheeses of 23 production batches after 26 weeks of ripening was mainly in line with the sensory scores obtained after 36 and 45 weeks of ripening (Supplementary Table S1). However, after 75 and 100 weeks of ripening, the Gouda cheeses with sensory score E had a lower average Z-score compared to those with sensory scores G and L. Moreover, 1, 6, 27, and 17 metabolites were significantly correlated with the sensory scores of Gouda cheeses of 36, 45, 75, and 100 weeks of ripening, respectively (Supplementary Table S2). These correlations often changed with the ripening time, as illustrated with the amino acids arginine and lysine that negatively correlated with the sensory scores after 45 weeks of ripening and positively after 75 and 100 weeks of ripening. The biogenic amines cadaverine, 2-phenylethylamine and putrescine, as well as 4-aminobutyric acid, had the strongest negative correlation with the sensory scores of cheeses of 75 and 100 weeks of ripening, whereas those compounds had no significant correlation with sensory scores after 36 and 45 weeks of ripening. At early ripening times, their concentrations were much lower and hence below their threshold values. Acetic acid, citrulline, gluconic acid, lactic acid, methional, phenylacetaldehyde, serine, and threonine were among the metabolites with a positive correlation with the sensory scores of the cheeses.

On species level, *Lc. cremoris* and *Loil. rennini* had a positive and negative correlation with the sensory scores of the cheeses after 75 weeks of ripening, respectively (Supplementary Table S3). At that ripening time, both LAB species were negatively correlated. *Loigolactobacillus rennini* was positively correlated with some biogenic amines and 4-aminobutyric acid; those compounds were negatively correlated with the sensory scores of the Gouda cheeses.

On ASV level, more significant correlations were found. In the case of *Lc. cremoris*, *Lacc. paracasei* and *T. halophilus*, opposite correlations were found within those species at different ripening times (Supplementary Table S4). The *T. halophilus* ASV cluster 1 had a strong negative correlation with the sensory scores of the 75-week cheeses and was positively correlated with *Loil. rennini*, whereas the *T. halophilus* ASV cluster 2 had no significant correlation with the sensory scores of the cheeses neither with the LAB species *Loil. rennini* (Figure 5).

## 4. Discussion

The present study deals with the investigation of batch-to-batch variations of a Gouda-type cheese produced on large scale in a European dairy factory, with special attention for the rotated starter culture mixtures applied, the ripening time, and the microbial and metabolite compositions of the cores and rinds of the cheese wheels. Although the commercial supplier of the mixed-strain starter cultures guaranteed that the starter culture mixtures A, B, and C used in the present study lead to similar end-products, significant differences were found between the Gouda A, B, and C cheeses produced, not only regarding their microbiome but also their metabolome. The main acidifiers, *Lc. lactis* and *Lc. cremoris*, did occur in all cheeses, albeit not in the same relative abundances, but species of the heterofermenter *Leuconostoc* did not, at least not to the same extent or according to the starter culture mixture used. The data of the present study further indicated that the dairy factory involved harbored a house microbiota that was mainly composed of *Lacc. paracasei*, *T. halophilus*, and *Loil. rennini*.

For the bacterial metagenetic analysis, a high-throughput, full-length 16S rRNA gene sequencing and ASV analysis was applied, which allowed accurate identifications at both species and strain level (Callahan et al., 2019). The big advantage of such a metagenetics approach lays in the single-nucleotide resolution, which even resulted in the detection of highly correlated, non-identical ASVs, probably belonging to one strain or cluster of strains. However, this deeper resolution also led to new challenges. For example, clusters with shared ASVs made further analysis less straightforward. Additionally, regardless of the precision, the use of only one gene might give a biased view on the bacterial diversity regarding subspecies level.

Most differences in microbiome and metabolome among the Gouda cheeses produced with the three different mixed-strain starter cultures applied were found after 36 weeks of ripening, as illustrated by the bacterial beta diversity and the scaled PCA of metabolites that grouped these cheese production batches according to the starter culture mixture used. At later ripening times, differences between the cheese production batches made with the same starter culture mixture increased and became more important than differences between the three groups of Gouda cheeses produced. This corroborated with a higher relative abundance of NSLAB that had a low diversity on species level across batches, but a striking diversity on ASV level. A similar situation has been described for a cheddar cheese factory, where cheese batches produced in different vats during the same day, or in the same vat during different days, have less than half of the NSLAB strains encountered as identical (Williams et al., 2002). The existence of a house microbiota and its role in the propagation of NSLAB in the factory and cheeses produced has been described frequently (Mounier et al., 2006; Bokulich and Mills, 2013a; Delcenserie et al., 2014; Calasso et al., 2016). Indeed, cheeses produced later during the day tend to have a higher bacterial diversity, suggesting a gradual accumulation of NSLAB in the production facility (O’Sullivan et al., 2015). This house microbiota was probably a main source of NSLAB contamination and associated variability for the 23 Gouda cheeses investigated.

The acidifying lactococci, *Lc. cremoris* and *Lc. lactis*, were the most abundant bacterial species in the Gouda cheeses produced, the former LAB species being present in higher relative abundances in the starter culture mixtures applied. The typical differences regarding salt tolerance and arginine conversion between these homofermentative LAB species were not always confirmed. For example, all Gouda cheese production batches had the same ASV profiles in the cores and rinds after 36 weeks of ripening, although it could be expected that the more salty rind would contain more ASVs of the more salt-tolerant *Lc. lactis*. Additionally, high arginine concentrations were not correlated with *Lc. cremoris*, although this species can not metabolize arginine (Schleifer et al., 1985). Yet, the higher arginine concentrations of the Gouda B cheeses could be linked with their slightly higher relative abundances of *Lc. cremoris*. Alternatively, in most cheese production batches, the relative abundance of *Lc. cremoris* decreased in favor of *Lc. lactis*, in particular between 45 and 100 weeks of ripening, which has to be ascribed to the faster speed of lysis of *Lc. cremoris* than *Lc. lactis* (Erkus et al., 2013; Fox et al., 2017).

Two ASVs of *Lc. cremoris*, *Lc. cremoris*_01 and *Lc. cremoris*_06, showed a high correlation with acetoin. However, acetoin is normally produced from citrate by *Lc. lactis* biovar diacetylactis, likely suggesting that the acetoin-producing strains lysed before 26 weeks of ripening. Alternatively, citrate-metabolizing and non-metabolizing *Lc. lactis* strains could share the same ASVs, which made it impossible to find meaningful correlations. Possibly, a substantial fraction of the *Lactococcus* reads was from cells in a VBNC state, as serine was one of the few amino acids positively correlated with *Lactococcus* and serine and methionine are particularly produced when *Lactococcus* enters a VBNC state (Ganesan et al., 2007).

In general, the different *Lactococcus* ASVs identified in the present study allowed to link each cheese production batch with its starter culture mixture applied, which can be of use for food authentication, although the differences in ASVs could not explain metabolic differences between the Gouda cheese production batches. However, it is well known that the *Lactococcus* genotype is often not in line with its phenotype (Tailliez et al., 1998; Fernández et al., 2011; Cavanagh et al., 2015), which has been reflected in the different reclassifications of *Lactococcus* species over time (Orla-Jensen, 1919; Garvie and Farrow, 1982; Schleifer et al., 1985; Li et al., 2021).

Considering the non-lactococci in the starter cultures used, a striking difference was the very low relative abundance of leuconostocs in the Gouda B cheeses, although they were present in the original starter culture mixtures applied. The Gouda A cheeses had a higher and approximately the same level of leuconostocs as the Gouda C cheeses. Yet, the presence of the *Leuc. pseudomesenteroides*_01 and *Leuc. pseudomesenteroides*_02 ASVs in the starter culture mixtures was lower than 0.008% (based on the total number of sequence reads). In general, undefined Gouda cheese starter cultures contain a low share of leuconostocs (Smid et al., 2014; Porcellato and Skeie, 2016; Frantzen et al., 2017). Overall, *Leuc. mesenteroides* was more abundant in the starter culture mixtures used, but it was marginally present in the Gouda cheeses produced compared to *Leuc. pseudomesenteroides*. In older literature and product information sheets, *Leuc. mesenteroides* subsp. *cremoris* and *Leuconostoc lactis* are considered as the main (sub)species in Gouda cheeses and mesophilic Gouda cheese starter cultures, but during the last 15 years, isolation of *Leuc. mesenteroides* subsp. *mesenteroides*, *Leuc. mesenteroides* subsp*. dextranicum*, and *Leuc. pseudomesenteroides* is more common (Frantzen et al., 2017). Of course, some *Leuc. pseudomesenteroides* isolates have also been wrongly identified as *Leuc. mesenteroides*, due to the use of solely partial 16S rRNA gene sequencing and/or phenotypical tests (Frantzen et al., 2017). Apart from a different effect on cheese metabolites, *Leuc. pseudomesenteroides* grows better and has a larger genome size compared to *Leuc. mesenteroides* subsp. *cremoris*, suggesting a higher survival potential during cheese ripening (Pedersen et al., 2016; Frantzen et al., 2017).

The three different ASV clusters of *Leuc. pseudomesenteroides* found in the Gouda cheeses of the present study correlated with either acidification and the lack of an efficient citrate metabolism (ASV clusters 1 and 3) or amino acid expulsion and flavor formation (ASV cluster 2). However, the lactococci associated with each *Leuconostoc* ASV cluster could be responsible for these differences as well. Indeed, leuconostocs are not believed to directly contribute to lactose fermentation (Ardö and Varming, 2010). Also, they lack cell envelope proteinases, making them dependent on peptide release from lactococci, and although they have only limited aminopeptidolytic activity, they can increase the concentrations of some amino acids extracellularly (Pedersen et al., 2016). Further, whereas the concentrations of most amino acids were lower in the Gouda B cheeses compared to the other ones, reflecting the lower relative abundance of leuconostocs, these cheeses contained the highest acetoin and 2,3-butanedione concentrations. This could be ascribed to the citrate-metabolizing *Lc. lactis* strains abundant or present in those cheeses, questioning the need of *Leuconostoc* for the production of these aroma compounds. Indeed, *Lc. lactis* biovar diacetylactis can even produce more acetoin compared to *Leuconostoc* (Starrenburg and Hugenholtz, 1991; Hugenholtz, 1993). However, the Gouda B cheeses with a low relative abundance of *Leuconostoc* contained more NSLAB, suggesting that inhibition of the growth of undesired bacteria seems to be an interesting contribution of *Leuconostoc*, an effect that has been described before (Martley and Crow, 1993). This effect was stronger for strains of the ASV cluster 1 in the Gouda C cheeses, compared to those of ASV cluster 2 in the Gouda A cheeses.

The increase of the relative abundances of the NSLAB species from 5 to 20% between 26 and 45 weeks of Gouda cheese ripening was in line with other culture-independent Gouda cheese studies (Porcellato and Skeie, 2016; Park et al., 2019). *Lacticaseibacillus paracasei* was the most abundant NSLAB species during the first 45 weeks of ripening, confirming previous reports (Van Hoorde et al., 2008; Kołakowski et al., 2012). It was represented by 10 of ASVs, differing between the Gouda cheese production batches and ripening times, but often occurring in a similar ratio, suggesting the presence of non-identical copies of the 16S rRNA gene within one strain. Possibly, the cheese manufacturing environment contained a high diversity of *Lacc. paracasei* and each production batch got inoculated with some strains that prevailed during ripening and contributed to the release of amino acids and the production of volatile organic compounds. In support of these beneficial properties, the use of *Lacc. paracasei* as adjunct culture for cheese production is well-known (Antonsson et al., 2003; Van Hoorde et al., 2010b; Stefanovic et al., 2018; Asahina et al., 2020; Bancalari et al., 2020; Randazzo et al., 2021).

*Tetragenococcus halophilus* was the most abundant NSLAB species in multiple Gouda cheeses after 75 and 100 weeks of ripening. This LAB species has been found culture-independently in Polish Oscypek cheese (Alegría et al., 2012), Spanish Cabrales and Manchego-type cheeses (Diaz et al., 2016; Botello-Morte et al., 2022), the rind of Gouda cheese (Salazar et al., 2018), and Irish hard cheese (only genus level; Quigley et al., 2012), but always at low relative abundances. Culture-dependently, it has been isolated from Mexican Cotija cheese (Morales et al., 2011), Brie de Meaux cheese (Unno et al., 2020), and Spanish blue-veined cheeses (Rodríguez et al., 2022). In these culture-dependent studies, *T. halophilus* has only been picked from media enriched with 2.5, 5.0, or 7.0% (m/v) NaCl. Besides a halophilic character, this LAB species displays an alkaliphilic behavior, as growth is better at pH 8.0 than at pH 7.0, whereas no growth occurs at pH 5.0 (Justé et al., 2012; Unno et al., 2020). Except for its salt dependency in agar media, its rare isolation could be ascribed to its prevalence in cheese rinds or long-ripened cheeses, which give lower counts with a culture-dependent analysis and fewer cells for proper DNA extraction with a culture-independent analysis.

The presence of *T. halophilus* in cheeses probably originates from the brine bath, as its occurrence in the latter was shown both culture-dependently and culture-independently (data not shown). Indeed, it has been found as a prevalent species in Belgian, Italian, and Danish brines (Marino et al., 2017; Haastrup et al., 2018; Vermote et al., 2018). One Italian brine shows a relative abundance of more than 50% (on sequence read basis) of *Tetragenococcus* and has a pH of 4.6, whereas the pH of the other brines varies between 4.8 and 5.7, suggesting that either growth is still possible at a pH lower than 5.0 or that no growth but survival occurs thanks to its salt tolerance. Since *T. halophilus* was already abundant in the Gouda cheese rinds of the present study after 36 weeks of ripening, it could likely proliferate and migrate from the brine to the cheese rind and finally to the core upon further cheese ripening. Furthermore, strong correlations were found between *T. halophilus* and the concentrations of free amino acids and short-chain fatty acids, which was in line with the proteolytic and lipolytic properties of isolates from Mexican cheeses (Morales et al., 2011). These properties could make *T. halophilus* interesting as an adjunct starter culture for (Gouda) cheese production. This LAB species is already used as primary starter culture for the production of oriental fermented salted food products, such as soy sauce, fish sauce, and shrimp paste (Justé et al., 2014). However, some *T. halophilus* strains harbor histidine and/or tyrosine decarboxylase genes, allowing histamine and/or tyramine production, respectively (Diaz et al., 2016; Chun et al., 2019). Alternatively, well-chosen strains would even lower biogenic amine formation, probably indirectly through the repression of other tetragenococci that produce biogenic amines (Udomsil et al., 2011; Kim et al., 2019), which supports the need to perform a genome sequencing and functional analysis approach of potential adjunct culture strains before their application.

Although *T. halophilus* was represented by more than 100 different ASVs in the Gouda cheeses examined, only two main ASV clusters occurred after 75 weeks of ripening, which confirmed its strain-dependent properties. Whereas the *T. halophilus* ASV cluster 1 was mainly found in cheeses with higher pH (which may have been caused by biogenic amine formation) and higher concentrations of short-chain fatty acids, the *T. halophilus* ASV cluster 2 was mainly found in cheeses with higher dry mass, reflecting the acid-sensitive and lipolytic character of strains of the former cluster and the osmotolerance of those of the latter one. In addition, the *T. halophilus* ASV cluster 1 strongly correlated with *Loil. rennini* and high and low concentrations of several biogenic amines and their precursor amino acids, respectively, after 75 weeks of ripening. The *T. halophilus* ASV cluster 2 was negatively correlated with histidine and positively correlated with histamine, suggesting the presence of a histidine decarboxylase gene that was expressed after 75 and 100 weeks of ripening. Indeed, histidine decarboxylase genes can be present in *T. halophilus* genomes, whereas other decarboxylase genes have not been found in the pangenome composed of 15 *T. halophilus* strains (Satomi et al., 2011; Chun et al., 2019).

Putrescine and cadaverine negatively impacted the sensory scores of the Gouda cheeses examined, whereas histamine did not. However, consumption of foods with a high histamine concentration can result in histamine poisoning (Özogul and Özogul, 2019). In the Gouda cheeses of the present study, the histamine concentrations were low up to 45 weeks of ripening, but they were around the Codex Alimentarius limit of 200 mg/kg (wet mass) established for fish and fish products after 75 and 100 weeks of ripening. Hence, because of a correlation of both ASV clusters 1 and 2 with the presence of several biogenic amines, a careful selection should be made when strains of these clusters are considered to be used as adjunct cultures. Indeed, in the case of the *T. halophilus* ASV cluster 1, the presence of biogenic amines could be related to the presence of *Loil. rennini*, and in the case of the *T. halophilus* ASV cluster 2, histamine production was most likely related to *T. halophilus*. However, the use of *T. halophilus* as adjunct culture could be made possible after curing of the plasmid encoding histamine production (Satomi et al., 2011; Botello-Morte et al., 2022).

The salt-tolerant *Loil. rennini*, which was prevalent in the Gouda cheese rinds after 36 weeks of ripening and in the cores of some cheese production batches after 75 and 100 weeks of ripening, was another prevalent NSLAB species. It was first isolated from rennet and held responsible for cracks and off-flavor defects in Dutch cheeses (Chenoll et al., 2006). It has also been found in Greek Kopanisti and Mana cheeses and in cheese brine (Asteri et al., 2009; Vermote et al., 2018). This LAB species, represented by a limited number of ASVs in the Gouda cheeses of the present study, correlated positively with different biogenic amines. Indeed, our investigation of the genome of a strain isolated from a 2-year Greek Kopanisti cheese (Kazou et al., 2017) revealed the presence of different decarboxylase genes, such as those coding for glutamate decarboxylase and ornithine decarboxylase, which yield 4-aminobutyric acid and putrescine, respectively. Moreover, a high correlation was found between *Loil. rennini* and a high pH after 75 weeks of ripening, likely to be ascribed to these decarboxylations.

The fungi found in the Gouda cheeses of the present study were in accordance with other reports on *Debaryomyces* as the most prevailing yeast in Gouda cheeses (Welthagen and Viljoen, 1998; Banjara et al., 2015). Whereas the latter studies did not differentiate between cheese cores and rinds, the present study showed that *Debaryomyces* was the most abundant yeast in the rind and *Saccharomyces* in the core of the Gouda cheeses produced. *Debaryomyces* has been found as a prevalent yeast in cheese brines and cheese production environments before (Mounier et al., 2006; Bokulich and Mills, 2013a; Vermote et al., 2018). Its higher salt tolerance and oxygen dependency than that of *Saccharomyces* (Sánchez et al., 2006) was reflected by the higher relative abundance of *Debaryomyces* in the Gouda cheese rinds and of *Saccharomyces* in the anaerobic cheese cores. The occurrence of yeasts in the Gouda cheeses of the present study may be linked with the production of different types of cheeses in the same dairy factory and their presence in cheese brines (Vermote et al., 2018). However, whereas some studies have mentioned yeast counts in Gouda cheeses up to 10^5^ CFU/g (Welthagen and Viljoen, 1998; Kołakowski et al., 2012), plating of some cheese samples of the present study on yeast-peptone-dextrose agar did not result in growth (data not shown), indicating that yeasts were present in very low numbers.

## 5. Conclusion

Batch-to-batch variation in mature Gouda cheeses was significant on microbial and metabolite level. Even when the rotated starter culture mixtures (A, B, and C) were considered to be similar, differences in microbial compositions and metabolite concentrations were found. A metagenetic approach based on high-throughput full-length 16S rRNA gene sequencing, accompanied with an ASV analysis, allowed differentiation of the bacterial communities on intra-species level. Starter culture mixture B did not result in prevailing leuconostocs in the corresponding cheeses, which led to a higher relative abundance of NSLAB. Hence, the presence of leuconostocs seemed to inhibit the growth of NSLAB, an effect that especially occurred in the Gouda C cheeses. Batch-to-batch differences generally increased with ripening time and became then less related to the starter culture mixture applied. Yet, metabolite differences between cores and rinds of the Gouda cheeses were found at all ripening times. The bacterial composition of the rinds at 36 weeks of ripening resembled those of the cores at 75 and 100 weeks of ripening. Among the NSLAB, which originate from the house microbiota that might vary, but will most probably be independent of the season given the constant temperature under which the factory is operating, *Lacc. paracasei* was most abundant until 45 weeks of ripening. This was taken over by *T. halophilus* and to a lesser extent *Loil. rennini*, which both could be responsible for biogenic amine formation and a bad flavor. Strong correlations of *T. halophilus* with desirable metabolites regarding aroma formation may make it a candidate adjunct culture. Finally, to increase Gouda cheese quality standardization, the use of only one starter culture mixture is proposed, together with a potential adjunct culture. Starter culture mixture rotation could still be applied, which each time should lead to another cheese type; for instance, starter culture mixture A should only be used for young Gouda cheeses, B for matured Gouda cheeses, and C for extra-matured Gouda cheeses. Also, the metagenetic ASV approach applied in the present study will allow differentiation of mixed-strain starter cultures across Gouda cheese producers in particular and contribute to food authentication in general.

## Data availability statement

The datasets presented in this study can be found in online repositories. The names of the repository/repositories and accession number(s) can be found at: https://www.ebi.ac.uk/ena, PRJEB58546.

## Author contributions

HD carried out the research and drafted the manuscript. SW and LDV supervised the research and revised and edited the manuscript. LDV was responsible for funding. All authors contributed to the article and approved the submitted version.

## Funding

This work was supported by the Research Council of the Vrije Universiteit Brussel (SRP7 and IOF3017 projects) and the Agency Flanders Innovation and Entrepreneurship (VLAIO).

## Conflict of interest

The authors declare that the research was conducted in the absence of any commercial or financial relationships that could be construed as a potential conflict of interest.

## Publisher’s note

All claims expressed in this article are solely those of the authors and do not necessarily represent those of their affiliated organizations, or those of the publisher, the editors and the reviewers. Any product that may be evaluated in this article, or claim that may be made by its manufacturer, is not guaranteed or endorsed by the publisher.
