# Supplementary material for: The rotation of primary starter culture mixtures results in batch-to-batch variations during Gouda cheese production
Source: Front Microbiol. 2023 Feb 16;14:1128394. doi: 10.3389/fmicb.2023.1128394 (PMC9978159; doi:10.3389/fmicb.2023.1128394)
Supplement: Supplementary file 1 [file Data_Sheet_1.docx]

Supplementary Material

The rotation of primary starter culture mixtures results in batch-to-batch variations during Gouda cheese production

Hannes Decadt, Stefan Weckx and Luc De Vuyst*

*** Correspondence:** Prof. Dr. ir. Luc De Vuyst: luc.de.vuyst@vub.be

# 1 Supplementary Tables

**Table S1.** Sensory scores for each Gouda cheese batch production at 26, 36, 45, 75, and 100 weeks of ripening. After 26 weeks of ripening, the cheeses were scored as less than average (L), good (G), or exceptionally good (E). For the other ripening times, the averages of the Z-scores were taken.

| Batch | 26 weeks | 36 weeks | 45 weeks | 75 weeks | 100 weeks |
| --- | --- | --- | --- | --- | --- |
| A1 | G | -0.36 | 0.86 | 0.15 | -0.11 |
| A2 | G | -0.37 | -0.24 | 0.69 | 0.40 |
| A3 | G | 0.32 | 0.27 | 0.28 | 0.36 |
| A4 | L | -0.22 | -0.12 | 0.39 | 0.18 |
| A5 | L | -0.34 | -0.12 | 0.22 | 0.02 |
| A6 | L | -0.68 | -0.85 | 0.17 | 0.07 |
| A7 | E | 0.82 | 0.41 | -0.23 | -0.99 |
| A8 | E | 0.10 | 0.16 | -2.04 | 0.20 |
| B1 | G | 0.75 | 0.38 | 0.95 | 0.50 |
| B2 | G | 0.10 | -0.17 | 0.10 | 0.58 |
| B3 | G | 0.06 | 0.02 | 0.35 | 0.18 |
| B4 | L | -0.07 | -0.49 | 0.24 | 0.03 |
| B5 | L | -0.19 | 0.27 | 0.50 | -0.43 |
| B6 | L | -0.37 | -0.73 | 0.51 | 0.46 |
| B7 | E | 0.78 | 0.31 | -1.69 | -0.08 |
| C1 | G | 0.05 | 0.19 | 0.37 | -0.16 |
| C2 | G | -0.11 | -0.19 | 0.06 | 0.05 |
| C3 | G | 0.14 | 0.36 | -0.29 | -1.20 |
| C4 | L | 0.36 | -0.05 | 0.42 | 0.17 |
| C5 | L | -0.76 | -0.10 | 0.28 | -0.19 |
| C6 | L | -0.19 | -0.15 | -0.15 | 0.05 |
| C7 | E | -0.10 | -0.09 | 0.58 | 0.05 |
| C8 | E | -0.33 | 0.08 | -1.85 | -1.06 |

**Table S2.** Pearson correlation coefficients between the metabolite concentrations and sensory scores of the Gouda cheeses from 23 different batch productions after 36, 45, 75, and 100 weeks of ripening. Only metabolites with correlations above 0.30 in absolute value are shown. Correlations represented in bold are significant. In the case that no correlation value is shown, the metabolite was not found at that specific ripening time. SCFA; short-chain fatty acid.

| Compound | 36 weeks | 45 weeks | 75 weeks | 100 weeks | Class | |  |
| --- | --- | --- | --- | --- | --- | --- | --- |
| 1-Butanol |  |  | -0.31 | -0.48 | Alcohol | | |
| 2,3-Butanediol | -0.36 | -0.34 | -0.26 | -0.35 | Alcohol | | |
| Phenylethyl alcohol | -0.32 | -0.07 | 0.12 | -0.06 | Alcohol | | |
| Benzaldehyde | 0.38 | 0.32 | -0.55 | -0.30 | Aldehyde | | |
| Hexanal | -0.26 | 0.47 |  |  | Aldehyde | | |
| Phenyl acetaldehyde | 0.07 | 0.00 | 0.44 | 0.28 | Aldehyde | | |
| Alanine | 0.20 | 0.13 | -0.01 | -0.55 | Amino acid | | |
| Arginine | -0.32 | -0.54 | 0.57 | 0.56 | Amino acid | |  |
| Asparagine | 0.34 | 0.12 | -0.21 | 0.18 | Amino acid | |  |
| Aspartic acid | 0.05 | 0.01 | -0.44 | -0.38 | Amino acid | |  |
| Citrulline | 0.00 | -0.16 | 0.62 | 0.56 | Amino acid | |  |
| 4-Aminobutyric acid | -0.37 | 0.17 | -0.67 | -0.65 | Amino acid | |  |
| Glycine | 0.38 | 0.15 | 0.36 | -0.06 | Amino acid | |  |
| Lysine | -0.19 | -0.46 | 0.69 | 0.48 | Amino acid |  |  |
| Ornithine | -0.21 | -0.43 | 0.46 | 0.12 | Amino acid |  |  |
| Phenylalanine | -0.12 | -0.13 | 0.61 | -0.17 | Amino acid |  |  |
| Proline | 0.10 | 0.33 | -0.45 | -0.56 | Amino acid |  |  |
| Serine | 0.13 | 0.01 | 0.51 | 0.57 | Amino acid |  |  |
| Threonine | 0.12 | 0.11 | 0.49 | 0.56 | Amino acid |  |  |
| Tryptophane | -0.10 | 0.04 | 0.55 | -0.55 | Amino acid |  |  |
| Tyrosine | 0.03 | 0.08 | 0.39 | -0.51 | Amino acid |  |  |
| Valine | -0.03 | 0.00 | -0.24 | -0.34 | Amino acid |  |  |
| Cadaverine | -0.15 | 0.22 | -0.65 | -0.57 | Biogenic amine | |  |
| 2-Phenylethylamine | -0.17 | 0.35 | -0.65 | -0.44 | Biogenic amine | |  |
| Putrescine | -0.15 | 0.21 | -0.60 | -0.56 | Biogenic amine | |  |
| Spermidine | 0.00 | -0.06 | 0.47 | 0.25 | Biogenic amine | |  |
| Spermine | -0.21 | -0.15 | 0.56 | 0.15 | Biogenic amine | |  |
| Tryptamine | -0.27 | 0.37 | -0.57 | -0.29 | Biogenic amine | |  |
| Tyramine | 0.15 | 0.49 | 0.19 | 0.20 | Biogenic amine | |  |
| δ-Dodecalactone | -0.18 | -0.28 | -0.38 | 0.13 | Ester |  |  |
| Ethyl dodecanoate |  |  | -0.58 | -0.4 | Ester |  |  |
| 2,3-Butanedione | 0.10 | 0.07 | -0.37 | -0.18 | Ketone |  |  |
| 2-Nonanone | 0.34 | -0.06 | -0.45 | -0.09 | Ketone |  |  |
| Acetoin | 0.18 | 0.00 | 0.39 | 0.26 | Ketone |  |  |
| D-Lactic acid | -0.17 | 0.15 | -0.31 | -0.33 | Organic acid |  |  |
| Gluconic acid | -0.33 | -0.09 | 0.46 | 0.33 | Organic acid |  |  |
| Glucuronic acid | 0.09 | -0.08 | 0.44 | 0.16 | Organic acid |  |  |
| Lactic acid | -0.15 | 0.17 | 0.44 | 0.35 | Organic acid |  |  |
| L-Lactic acid | 0.17 | -0.15 | 0.31 | 0.33 | Organic acid |  |  |
| Malic acid | 0.34 | -0.02 | 0.26 | 0.03 | Organic acid |  |  |
| Orotic acid | 0.00 | 0.47 | -0.09 | -0.16 | Organic acid |  |  |
| Oxalic acid | 0.38 | -0.19 | 0.30 | 0.12 | Organic acid |  |  |
| Pyruvic acid | -0.22 | -0.05 | 0.14 | 0.32 | Organic acid |  |  |
| Succinic acid | -0.11 | -0.11 | 0.40 | 0.06 | Organic acid |  |  |
| Uric acid |  | -0.10 | 0.08 | -0.57 | Organic acid |  |  |
| Tetramethylpyrazine | 0.13 | 0.17 | -0.49 | -0.31 | Pyrazine |  |  |
| Acetic acid | -0.38 | -0.23 | 0.38 | 0.63 | SCFA |  |  |
| Butyric acid | -0.26 | 0.03 | -0.3 | -0.15 | SCFA |  |  |
| Hexanoic acid | -0.12 | 0.00 | -0.33 | -0.17 | SCFA |  |  |
| Propionic acid | -0.21 | 0.16 | -0.38 | -0.62 | SCFA |  |  |
| Dimethyl sulfone | 0.06 | 0.10 | -0.46 | -0.17 | Sulphur |  |  |
| Methional | 0.43 | 0.02 | 0.38 | 0.27 | Sulphur |  |  |

**Table S3.** Pearson correlation coefficients between the major bacterial species and sensory scores of the Gouda cheeses from 23 different batch productions after 36, 45, 75, and 100 weeks of ripening. Correlations represented in bold are significant.

| Species | 36 weeks | 45 weeks | 75 weeks | 100 weeks |
| --- | --- | --- | --- | --- |
| *Lacticaseibacillus paracasei* | 0.21 | 0.16 | -0.04 | -0.13 |
| *Lacticaseibacillus rhamnosus* | -0.17 | -0.33 | 0.16 | 0.23 |
| *Lactiplantibacillus plantarum* | -0.18 | -0.41 | 0.20 | 0.17 |
| *Lactococcus cremoris* | 0.36 | -0.32 | 0.50 | -0.34 |
| *Lactococcus lactis* | -0.02 | 0.04 | 0.20 | 0.14 |
| *Lactococcus laudensis* | -0.01 | 0.05 | 0.10 | -0.07 |
| *Leuconostoc mesenteroides* | -0.03 | -0.34 | 0.19 | 0.12 |
| *Leuconostoc pseudomesenteroides* | -0.19 | 0.25 | -0.13 | -0.25 |
| *Loigolactobacillus rennini* | -0.20 | 0.13 | -0.76 | -0.04 |
| *Paucilactobacillus oligofermentans* | 0.02 | -0.06 | 0.08 | 0.06 |
| *Propionibacterium freudenreichii* | 0.36 | 0.14 | 0.21 | -0.03 |
| *Staphylococcus equorum* | -0.07 | 0.28 | 0.05 | 0.14 |
| *Streptococcus thermophilus* | 0.25 | 0.18 | 0.11 | -0.05 |
| *Tetragenococcus halophilus* | -0.11 | 0.22 | -0.40 | 0.16 |

**Table S4.** Pearson correlation coefficients between the bacterial amplicon sequence variants (ASVs) and sensory scores of the Gouda cheeses from 23 different batch productions after 36, 45, 75, and 100 weeks of ripening. Only the most abundant ASVs (more than 50 reads in at least two cases) are shown. Correlations represented in bold are significant. No correlation means that the ASV was not detected at that specific time point.

| ASV | 36 weeks | 45 weeks | 75 weeks | 100 weeks |
| --- | --- | --- | --- | --- |
| *Lacticaseibacillus paracasei*_01 | -0.12 | -0.01 | 0.11 | -0.10 |
| *Lacticaseibacillus paracasei*_02 | 0.27 | 0.02 | 0.06 | -0.07 |
| *Lacticaseibacillus paracasei*_03 | -0.14 | -0.06 | 0.15 | -0.14 |
| *Lacticaseibacillus paracasei*_04 | 0.38 | 0.20 | -0.46 | 0.08 |
| *Lacticaseibacillus paracasei*_05 | 0.35 | 0.32 | -0.41 | 0.01 |
| *Lacticaseibacillus paracasei*_06 | 0.38 | 0.24 | -0.46 | 0.01 |
| *Lacticaseibacillus paracasei*_07 | 0.44 | 0.01 | 0.02 | 0.06 |
| *Lacticaseibacillus paracasei*_08 | 0.18 | 0.08 | -0.31 | 0.15 |
| *Lacticaseibacillus paracasei*_09 | -0.25 | 0.10 | -0.16 | -0.58 |
| *Lacticaseibacillus paracasei*_10 | -0.06 | 0.00 | 0.06 | 0.14 |
| *Lacticaseibacillus paracasei*_11 | 0.41 | 0.22 | 0.03 | 0.08 |
| *Lacticaseibacillus paracasei*_12 | -0.28 | 0.01 | -0.08 | -0.61 |
| *Lacticaseibacillus paracasei*_13 | -0.15 | 0.53 | -0.21 | -0.07 |
| *Lacticaseibacillus paracasei*_15 | -0.15 | 0.49 | -0.20 | -0.04 |
| *Lacticaseibacillus paracasei*_17 | -0.17 | 0.49 | -0.20 | -0.09 |
| *Lacticaseibacillus paracasei*_18 | 0.18 | -0.13 | 0.06 | 0.02 |
| *Lacticaseibacillus paracasei*_19 | -0.28 | -0.02 | -0.06 | -0.56 |
| *Lacticaseibacillus paracasei*_22 | 0.07 |  |  | -0.05 |
| *Lacticaseibacillus paracasei*_24 | -0.31 | 0.31 | -0.14 | -0.57 |
| *Lacticaseibacillus paracasei*_25 | -0.02 | -0.13 | -0.56 | 0.04 |
| *Lacticaseibacillus paracasei*_26 | 0.13 | 0.33 | -0.31 | -0.10 |
| *Lacticaseibacillus paracasei*_28 | -0.05 | -0.07 | 0.06 |  |
| *Lacticaseibacillus paracasei*_30 | -0.02 | -0.28 |  | 0.03 |
| *Lacticaseibacillus rhamnosus*_01 | -0.16 |  | 0.14 | 0.23 |
| *Lactiplantibacillus plantarum*_01 | -0.19 | -0.40 | 0.18 | 0.15 |
| *Lactiplantibacillus plantarum*_02 | -0.18 | -0.41 | 0.18 | 0.19 |
| *Lactiplantibacillus plantarum*_03 | -0.19 | -0.41 | 0.18 | 0.16 |
| *Lactiplantibacillus plantarum*_04 | -0.19 | -0.41 | 0.17 | 0.19 |
| *Lactiplantibacillus plantarum*_05 | -0.19 | -0.41 | 0.17 | 0.16 |
| *Lactiplantibacillus plantarum*_06 | -0.03 |  | 0.10 | 0.10 |
| *Lactiplantibacillus plantarum*_07 | -0.18 |  |  |  |
| *Lactiplantibacillus plantarum*_08 | -0.02 |  | 0.10 | 0.09 |
| *Lactiplantibacillus plantarum*_11 | -0.18 |  | 0.14 |  |
| *Lactococcus cremoris*_01 | 0.32 | -0.30 | 0.58 | -0.29 |
| *Lactococcus cremoris*_02 | 0.25 | -0.18 | 0.25 | -0.44 |
| *Lactococcus cremoris*_03 | -0.13 | 0.11 | 0.14 | 0.20 |
| *Lactococcus cremoris*_04 | 0.00 | -0.24 | 0.24 | -0.46 |
| *Lactococcus cremoris*_05 | 0.05 | -0.43 | 0.36 | -0.21 |
| *Lactococcus cremoris*_06 | 0.37 | -0.22 | 0.32 | 0.08 |
| *Lactococcus cremoris*_07 | 0.19 | 0.24 | 0.44 | -0.15 |
| *Lactococcus cremoris*_08 | -0.02 | -0.23 | 0.15 | -0.58 |
| *Lactococcus cremoris*_09 | -0.13 | 0.27 | 0.18 |  |
| *Lactococcus cremoris*_10 | 0.16 | -0.06 | 0.25 | -0.18 |
| *Lactococcus cremoris*_12 | -0.02 | -0.19 | 0.19 | -0.46 |
| *Lactococcus cremoris*_13 | -0.01 | -0.41 | 0.31 | -0.26 |
| *Lactococcus cremoris*_14 | 0.29 | -0.02 | 0.17 | -0.05 |
| *Lactococcus cremoris*_15 | 0.06 | -0.32 | 0.18 | -0.45 |
| *Lactococcus cremoris*_16 | 0.06 | 0.44 | 0.06 | 0.04 |
| *Lactococcus lactis*_01 | -0.02 | 0.04 | 0.21 | 0.14 |
| *Lactococcus lactis*_02 | 0.00 | 0.00 | -0.20 | -0.10 |
| *Lactococcus lactis*_03 | 0.09 | 0.26 | 0.01 | 0.19 |
| *Lactococcus lactis*_04 | 0.41 | 0.10 | 0.16 | 0.10 |
| *Lactococcus lactis/cremoris*_01 | -0.09 | -0.26 | 0.49 | -0.41 |
| *Lactococcus lactis/cremoris*_02 | -0.11 | -0.24 | 0.57 | -0.39 |
| *Lactococcus lactis/cremoris*_03 | -0.05 | -0.24 | 0.42 | -0.50 |
| *Lactococcus lactis/cremoris*_04 | -0.19 | -0.18 | 0.51 | -0.36 |
| *Lactococcus lactis/cremoris*_05 | -0.04 | -0.14 | 0.29 | -0.41 |
| *Lactococcus lactis/cremoris*_06 | 0.01 | -0.02 | 0.15 | -0.30 |
| *Lactococcus lactis/cremoris*_07 | 0.17 | -0.40 | 0.40 | 0.09 |
| *Lactococcus lactis/cremoris*_08 | -0.03 | -0.20 | 0.46 | -0.36 |
| *Lactococcus lactis/cremoris*_09 | 0.09 |  |  |  |
| *Lactococcus lactis/cremoris*_10 | 0.12 | -0.14 | 0.23 | 0.05 |
| *Lactococcus lactis/cremoris*_11 | 0.04 | 0.21 | -0.71 | 0.05 |
| *Lactococcus laudensis*_01 | -0.05 | 0.36 | -0.01 | -0.06 |
| *Lactococcus laudensis*_02 | 0.06 | -0.25 | 0.14 | -0.15 |
| *Lactococcus laudensis*_04 | 0.04 | 0.54 | 0.06 |  |
| *Leuconostoc mesenteroides*_01 | -0.05 | -0.15 | 0.07 | 0.15 |
| *Leuconostoc mesenteroides*_02 | 0.03 | -0.36 | 0.23 | -0.02 |
| *Leuconostoc pseudomesenteroides*_01 | -0.19 | 0.24 | -0.14 | -0.29 |
| *Leuconostoc pseudomesenteroides*_02 | -0.17 | 0.35 | -0.12 | -0.26 |
| *Leuconostoc pseudomesenteroides*_03 | 0.04 | -0.01 | 0.06 | 0.13 |
| *Loigolactobacillus rennini*_01 | -0.20 | 0.15 | -0.75 | -0.05 |
| *Loigolactobacillus rennini*_02 | -0.22 | 0.16 | -0.76 | -0.06 |
| *Loigolactobacillus rennini*_03 | -0.20 | 0.14 | -0.75 | -0.05 |
| *Loigolactobacillus rennini*_04 | -0.17 | -0.07 | -0.71 | 0.22 |
| *Loigolactobacillus rennini*_05 | -0.10 | -0.07 | -0.69 | 0.23 |
| *Loigolactobacillus rennini*_06 | -0.10 | -0.07 | -0.74 | 0.23 |
| *Paucilactobacillus oligofermentans*_01 | 0.00 | -0.06 | 0.08 | 0.07 |
| *Propionibacterium freudenreichii*_01 | 0.38 | 0.15 | 0.22 | -0.03 |
| *Sporolactobacillus sp.*_01 |  | -0.28 |  | 0.25 |
| *Sporolactobacillus sp.*_02 |  | -0.28 |  | 0.10 |
| *Sporolactobacillus sp.*_03 |  | -0.28 |  | 0.25 |
| *Sporolactobacillus sp.*_04 |  | -0.28 |  |  |
| *Staphylococcus equorum*_01 | -0.05 | 0.33 | 0.05 | 0.14 |
| *Staphylococcus equorum*_06 | -0.16 | 0.10 | -0.02 | 0.12 |
| *Tetragenococcus halophilus*_001 | -0.14 | 0.15 | -0.65 | 0.15 |
| *Tetragenococcus halophilus*_002 | -0.16 | -0.04 | -0.67 | 0.11 |
| *Tetragenococcus halophilus*_003 | -0.17 | -0.02 | -0.66 | 0.13 |
| *Tetragenococcus halophilus*_004 | -0.17 | -0.04 | -0.71 | 0.17 |
| *Tetragenococcus halophilus*_005 | -0.18 | -0.05 | -0.75 | 0.28 |
| *Tetragenococcus halophilus*_006 | -0.06 | 0.26 | -0.14 | 0.18 |
| *Tetragenococcus halophilus*_007 | -0.06 | 0.24 | -0.12 | 0.16 |
| *Tetragenococcus halophilus*_008 | -0.08 | 0.25 | -0.11 | 0.17 |
| *Tetragenococcus halophilus*_009 | -0.08 | 0.25 | -0.16 | 0.14 |
| *Tetragenococcus halophilus*_010 | -0.08 | 0.24 | -0.12 | 0.14 |
| *Tetragenococcus halophilus*_011 | 0.02 | 0.24 | -0.60 | -0.17 |
| *Tetragenococcus halophilus*_012 | -0.05 | 0.23 | -0.59 | -0.16 |
| *Tetragenococcus halophilus*_013 | 0.00 | -0.04 | -0.61 | 0.01 |
| *Tetragenococcus halophilus*_014 | 0.04 | 0.21 | -0.59 | -0.06 |
| *Tetragenococcus halophilus*_015 | -0.10 |  | -0.46 | -0.08 |
| *Tetragenococcus halophilus*_016 | -0.05 | 0.29 | 0.03 | 0.04 |
| *Tetragenococcus halophilus*_017 |  |  | 0.08 | -0.18 |
| *Tetragenococcus halophilus*_018 |  |  | -0.39 | 0.09 |
| *Tetragenococcus halophilus*_019 |  |  | 0.09 | 0.20 |
| *Tetragenococcus halophilus*_020 |  |  | -0.47 |  |
| *Tetragenococcus halophilus*_021 |  |  | -0.56 | 0.05 |
| *Tetragenococcus halophilus*_022 | -0.04 | 0.27 | 0.27 | 0.45 |
| *Tetragenococcus halophilus*_023 | -0.09 | 0.20 | -0.09 | -0.10 |
| *Tetragenococcus halophilus*_024 | 0.07 | 0.30 | 0.25 | -0.23 |
| *Tetragenococcus halophilus*_025 | -0.10 |  | 0.17 | 0.15 |
| *Tetragenococcus halophilus*_026 | -0.04 | 0.28 | 0.27 | 0.46 |
| *Tetragenococcus halophilus*_027 | 0.08 | 0.03 | -0.09 | -0.12 |
| *Tetragenococcus halophilus*_028 |  |  | -0.59 | -0.10 |
| *Tetragenococcus halophilus*_029 | -0.07 | 0.16 | -0.02 | -0.13 |
| *Tetragenococcus halophilus*_030 | -0.10 |  |  | 0.07 |
| *Tetragenococcus halophilus*_031 |  |  | -0.45 | -0.28 |
| *Tetragenococcus halophilus*_032 |  |  | -0.57 | -0.05 |
| *Tetragenococcus halophilus*_033 | -0.08 |  | 0.05 | -0.03 |
| *Tetragenococcus halophilus*_035 | -0.08 |  | 0.08 | 0.19 |
| *Tetragenococcus halophilus*_036 | 0.54 | -0.19 | 0.00 | -0.12 |
| *Tetragenococcus halophilus*_037 |  |  | -0.56 | 0.22 |
| *Tetragenococcus halophilus*_038 | -0.10 |  | -0.45 | 0.34 |
| *Tetragenococcus halophilus*_039 |  |  | 0.19 | 0.23 |
| *Tetragenococcus halophilus*_041 | -0.08 |  | -0.37 | 0.02 |
| *Tetragenococcus halophilus*_042 |  |  | -0.25 | -0.06 |
| *Tetragenococcus halophilus*_043 |  |  | -0.46 | -0.06 |
| *Tetragenococcus halophilus*_044 |  |  | -0.32 | -0.03 |
| *Tetragenococcus halophilus*_045 | -0.25 | -0.01 | -0.56 | -0.02 |
| *Tetragenococcus halophilus*_046 | 0.09 | 0.29 | -0.04 | -0.18 |
| *Tetragenococcus halophilus*_047 |  |  |  | 0.00 |
| *Tetragenococcus halophilus*_048 | -0.08 |  |  | 0.08 |
| *Tetragenococcus halophilus*_049 |  |  | -0.56 | 0.21 |
| *Tetragenococcus halophilus*_050 |  |  |  | 0.18 |
| *Tetragenococcus halophilus*_052 |  |  | 0.08 | 0.18 |
| *Tetragenococcus halophilus*_053 | 0.07 |  | -0.73 | 0.20 |
| *Tetragenococcus halophilus*_054 |  |  |  | 0.07 |
| *Tetragenococcus halophilus*_056 |  |  |  | 0.03 |
| *Tetragenococcus halophilus*_058 |  |  |  | 0.06 |
| *Tetragenococcus halophilus*_068 |  |  |  | 0.21 |

# 2 Supplementary Figures

**Figure S1.** **(Top)** Average alpha diversity, expressed as inverse Simpson index, of the bacterial species compositions of Gouda cheeses made with three different mixed-strain starter cultures (A, red; B, green; C, blue) for the cores after 26, 31, 36, 45, 75, and 100 weeks of ripening and the rinds after 36 weeks of ripening. **(Bottom)** Beta diversity, expressed as *p*-value, of the bacterial species compositions of the Gouda A and B cheeses (brown), the Gouda A and C cheeses (purple), and the Gouda B and C cheeses (turquoise). High and low *p*-values indicate a high and low similarity, respectively, between the Gouda cheeses made with two different starter culture mixtures.





**Figure S2.** Principal component analysis (PCA) of the bacterial species compositions in the Gouda cheese cores after 26 (yellow), 31 (green), 36 (light blue), 45 (purple), 75 (red), and 100 (orange) weeks of ripening and in the rinds after 36 (dark blue) weeks of ripening. The Gouda A cheeses are indicated with circles, the Gouda B cheeses with triangles, and the Gouda C cheeses with squares. The black arrows indicate the six highest loadings of the PCA. C, *Lactococcus cremoris*; L, *Lactococcus lactis*; M, *Leuconostoc pseudomesenteroides*; P, *Lacticaseibacillus paracasei*; R, *Loigolactobacillus rennini*; T, *Tetragenococcus halophilus.*

**Figure S3.** Spearman correlations between the relative abundances of the major bacterial species found in the Gouda cheeses from 23 different batch productions at all ripening times examined. Significant correlations are represented in bold.


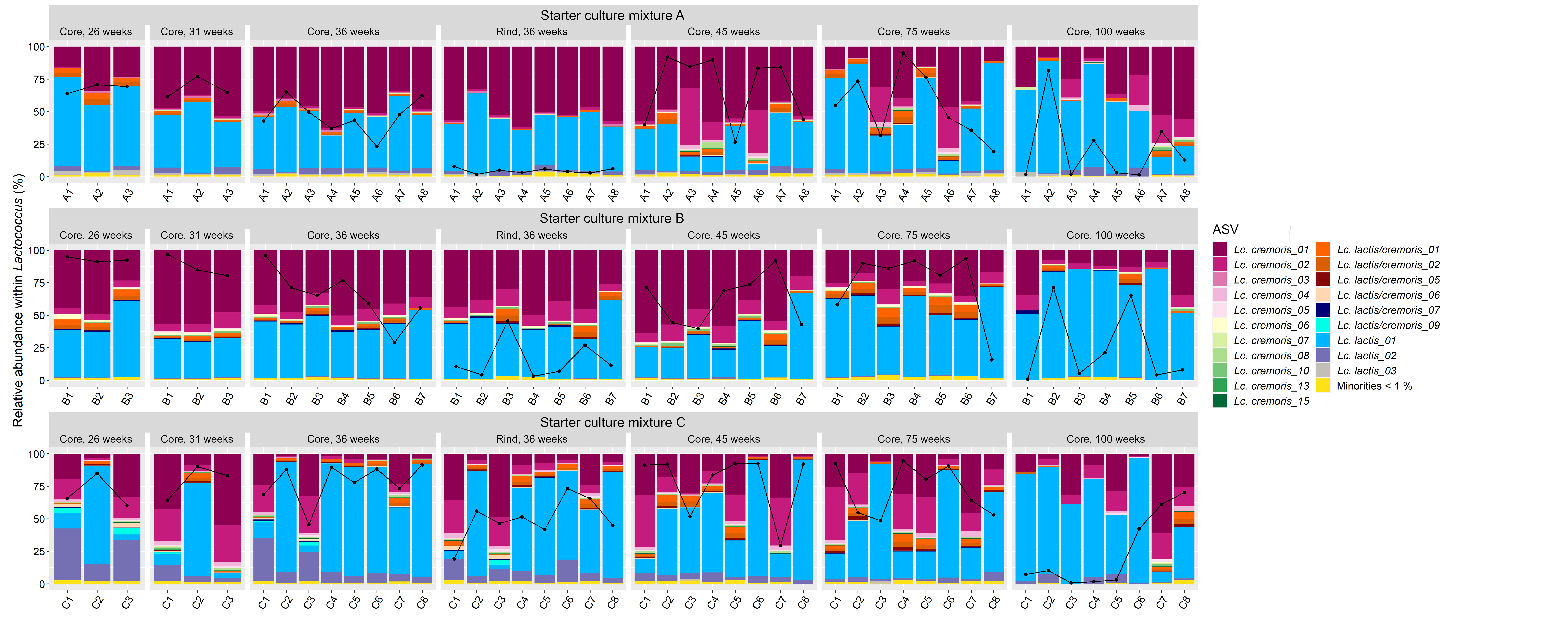





**Figure S4.** Relative abundances of the amplicon sequence variants (ASVs), based on the full-length 16S rRNA gene of the starter lactic acid bacteria species *Lactococcus cremoris* and *Lactococcus lactis* **(top)** and *Leuconostoc* **(bottom)**, identified in Gouda cheeses from 23 different batch productions made with three different mixed-strain starter cultures (A, B, and C) after 26, 31, 36, 45, 75, and 100 weeks of ripening (cores) and 36 weeks of ripening (rinds). The numbers indicate the cheeses of the 23 different batch productions. The black line indicates the total percentage of *Lc. cremoris* and *Lc. lactis* sequence reads (top) and *Leuconostoc* sequence reads (bottom) in each cheese. ASVs belonging to *Lc. cremoris* or *Lc. lactis* that could not be assigned to one of these two species are indicated as *Lc. lactis/cremoris*.





**Figure S5.** Taxonomic assessment of the *Leuconostoc* isolates from the commercial starter culture mixtures A, B, and C on amplicon sequence variant (ASV) level, based on the full-length 16S rRNA gene. The presence of two ASVs for an isolate indicates non-identical copies of the 16S rRNA gene in the genome.


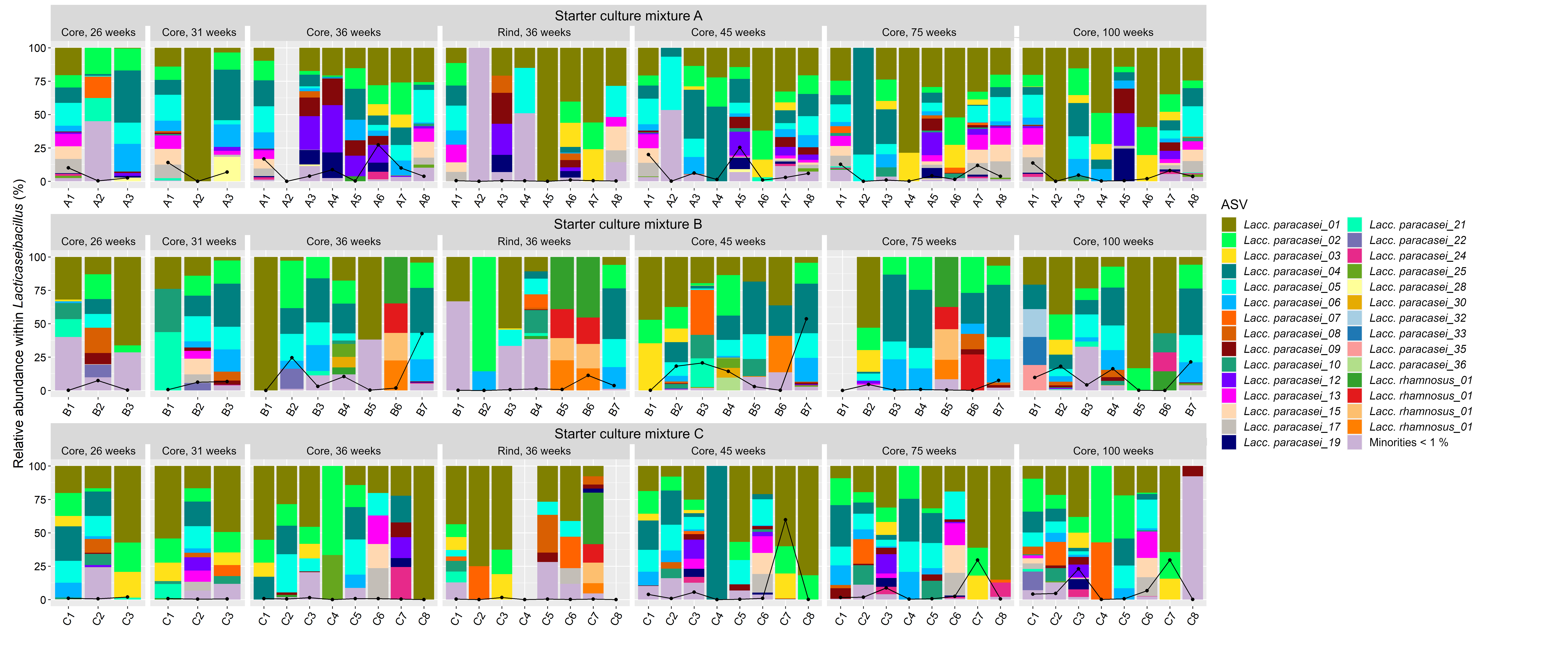





**Figure S6.** Relative abundance of the amplicon sequence variants (ASVs), based on the full-length 16S rRNA gene of the non-starter lactic acid bacteria genera *Lacticaseibacillus* **(top)** and *Lactiplantibacillus* **(bottom)** identified in Gouda cheeses from 23 different batch productions made with three different mixed-strain starter cultures (A, B, and C), after 26, 31, 36, 45, 75, and 100 weeks of ripening (cores) and 36 weeks of ripening (rinds). The numbers indicate the cheeses of the 23 different batch productions. The black line indicates the total percentage of *Lacticaseibacillus* sequence reads (top) and *Lactiplantibacillus* sequence reads (bottom) in each cheese.
